# SEAS (Scientific Exercises Approach to Scoliosis): a modern and effective evidence based approach to physiotherapic specific scoliosis exercises

**DOI:** 10.1186/s13013-014-0027-2

**Published:** 2015-02-05

**Authors:** Michele Romano, Alessandra Negrini, Silvana Parzini, Marta Tavernaro, Fabio Zaina, Sabrina Donzelli, Stefano Negrini

**Affiliations:** ISICO (Italian Scientific Spine Institute), Via Roberto Bellarmino 13, 20141 Milan, Italy; University of Brescia, Brescia, Italy; IRCCS Don Gnocchi, Milan, Italy

## Abstract

**Background:**

SEAS is the acronym for “Scientific Exercise Approach to Scoliosis”, a name related to the continuous changes of the approach based on results published in the literature.

**Rehabilitation program:**

SEAS is an individualized exercise program adapted to all situations of conservative treatment of scoliosis: stand-alone in low-medium degree curves during growth to reduce the risk of bracing; complimentary to bracing in medium-high degree curves during growth, with the aim to increase correction, prepare weaning, and avoid/reduce side-effects; for adults either progressing or fused, to help stabilising the curve and reduce disability.

SEAS is based on a specific active self-correction technique performed without external aid, and incorporated in functional exercises. Evaluation tests guide the choice of the exercises most appropriate to the individual patient.

Improvement of the stability of the spine in active self-correction is the primary objective of SEAS. SEAS exercises train neuromotor function so to stimulate by reflex a self-corrected posture during the activities of daily life.

SEAS can be performed as an outpatient (two/three times a week 45 for minutes) or as a home program to be performed 20 minutes daily. In the last case, expert physiotherapy sessions of 1.5 hours every three months are proposed.

**Results:**

Different papers, including a randomized controlled trial (2014), published over the past several years, documented the efficacy of the SEAS approach applied in the various phases of scoliosis treatment in reducing Cobb angle progression and the need to wear a brace.

**Conclusions:**

SEAS is an approach to scoliosis exercise treatment with a strong modern neurophysiological basis, to reduce requirements for patients and possibly the costs for families linked to the frequency and intensity of treatment and evaluations. Therefore, SEAS allows treating a large number of patients coming from far away. Even if SEAS appears simple by requiring less physiotherapist supervision and by using fewer home exercises prescribed at a lower dose than some of the other scoliosis-specific exercise approaches, real expertise in scoliosis, exercises, and patient and family management is required. The program has no copyrights, and teachers are being trained all over the world.

## Introduction

SEAS is the acronym for “Scientific Exercise Approach to Scoliosis”, a name related to the continuous changes of the approach, based on results published in the literature. In fact, a characteristic of Science is, that what is known and believed true today is going to change in time. Consequently, the adjective Scientific has been usedas the approach has already evolved since its introduction and will change in the future as new relevant scientific knowledge becomes available.

## History

The SEAS approach is the result of a history that began as long ago as the early 1960s, when in Vigevano (Italy) Antonio Negrini and Nevia Verzini founded a scoliosis center that become later the “Centro Scoliosi Negrini” (CSN). The founders of CSN developed a treatment in which exercises were directed toward therapeutic objectives specifically derived from the data provided by scientific research methods: for this reason, they were among the founders of the Italian Study Group of Scoliosis that started from 1978 to systematically search the international literature to find the best scientific papers related to conservative treatment of scoliosis. They exchanged information and experiences with different scoliosis centers in different European coutries (Switzerland, Sweden, France). In particular, the CSN began a collaborative effort in the study and research of scoliosis with the “Centre des Massues” in Lyon, France, which in those days was considered one of Europe’s most prestigious centres for scoliosis treatment. In those years, the prestige was based on the number of patients who came from abroad and on scientific evidence of conservative treatment based on the use of braces and surgery. The evidence of physiotherapeutic treatment had not yet been produced, even if the first study showing the effectiveness of exercises for AIS was produced by the Lyon School and also included data of more than 100 patients of the CSN [1].

The SEAS approach originates from the Lyon approach [2,3], some of the base characteristics previously published for the Lyon approach have been retained in SEAS:

- increasing patient’s awareness of the deformity
- emphasising an independent auto-correction by the patient
- use of exercises in which balance reactions are elicited
- ensuring that the patient wears a brace for at least some of the exercises, so as to use the orthotic as a gym instrument; for example, to control the self-correction in cases where the patient is unable to do this properly and without help while performing the exercises, and to have a greater effect on the modelling of aesthetics.

The most important differences gradually developed by SEAS, that made it different from the Lyon approach, include:

- active three-dimensional self-correction instead of the former auto-elongation [4,5]
- spinal stabilisation concept according to the actual physiotherapeutic literature [6,7]
- research of an automatic correct reflex response, namely, a subconscious self-correction which should help to obtain the better integration in the daily life [8].
- focus on the cognitive-behavioural approach of the patient to increase compliance to treatment [9]
- variability of exercises stimuli instead of absolute repetitive precision of movements, according to modern neurophysiologic knowledge [10,11]

## Theoretical principles

The main aim of SEAS is to reverse the Stokes vicious cycle, so, the abnormal loading created by the scoliosis with an asymmetric growth leading to worsening of curves that will lead to further asymmetric growth due to increased asymmetrical loading [12,13].

The SEAS works with a specific difference with bracing: in fact, while an orthotic device can change continuously the posture of the patient making it somehow fixed [14-29], exercises can only determine behavioural and automatic changes of movement and posture through different motor control strategies [8,17-19]. This is particularly important for a bodily system like the trunk and spine, that has been demonstrated to be driven more by automatic, feed-forward schemes than voluntary control [8]. Moreover, as shown by Stokes, active movement is more effective than passive positioning in determining changes of spinal deformity [20]: this is one of the reasons for an active exercises approach as a stand-alone treatment or coupled with bracing according to the so-called active bracing principle [21,22]. In this theoretical framework, SEAS exercises are based on autocorrection and stabilisation.

Another very important specific basis for scoliosis exercises has recently been described by Smania and co-authors [8], that focused on some neurophysiological peculiarities of AIS relevant for rehabilitation. They include: organization of patterns of trunk muscle recruitment, neural structures serving axial and arm muscle control, and relevance of cognitive systems in the building of body schema and mapping of spatial coordinates. Trunk control is generally carried out by means of very fast, feed-forward or feed-back driven patterns of muscle activation; these are very deep in our neurological control system: consequently it is very difficult to modify them through a specific voluntary training.

## SEAS active self-correction

Self-correction can be defined as the search for the best possible alignment the patient can achieve in the three spatial planes. Three-dimensional self-correction is considered one of the most important elements for the organization of conservative treatment. The SOSORT (International Society on Scoliosis Orthopaedic and Rehabilitation Treatment) consensus in 2005 [23] attributes to the “3D Self-correction ” first place in the ranking of important elements to be included in the exercises [19]. Even if the modality of obtaining it is not the same, all widely used international approaches based on exercises (like Schroth [24,25], Dobomed [26], Side Shift [27], FITS [28]) systematically include self-correction.

The purpose of SEAS exercises is to train an automatic response to the achievement of a more correct position [29] so as to stimulate the maintenance of a three-dimensionally corrected posture during the activities of daily life. According to the SEAS approach, achieving an “indirect” self-correction using external aids does not allow the achievement of the purpose on which this concept is based. For this reason, active self-correction, according to the SEAS approach, must be done in a “direct” manner, i.e. without external aid. Thus it is necessary to use the intrinsic muscles of the spine as much as possible and not be aided by supports, traction or girdles. The exercises propose a fundamental conceptual shift. It is not the “best passive alignment” that is sought but instead the “best functional stimulation of an independent contraction of muscles trained for the search of the best alignment of the spine” [8,29,30].

**Figure 1:**
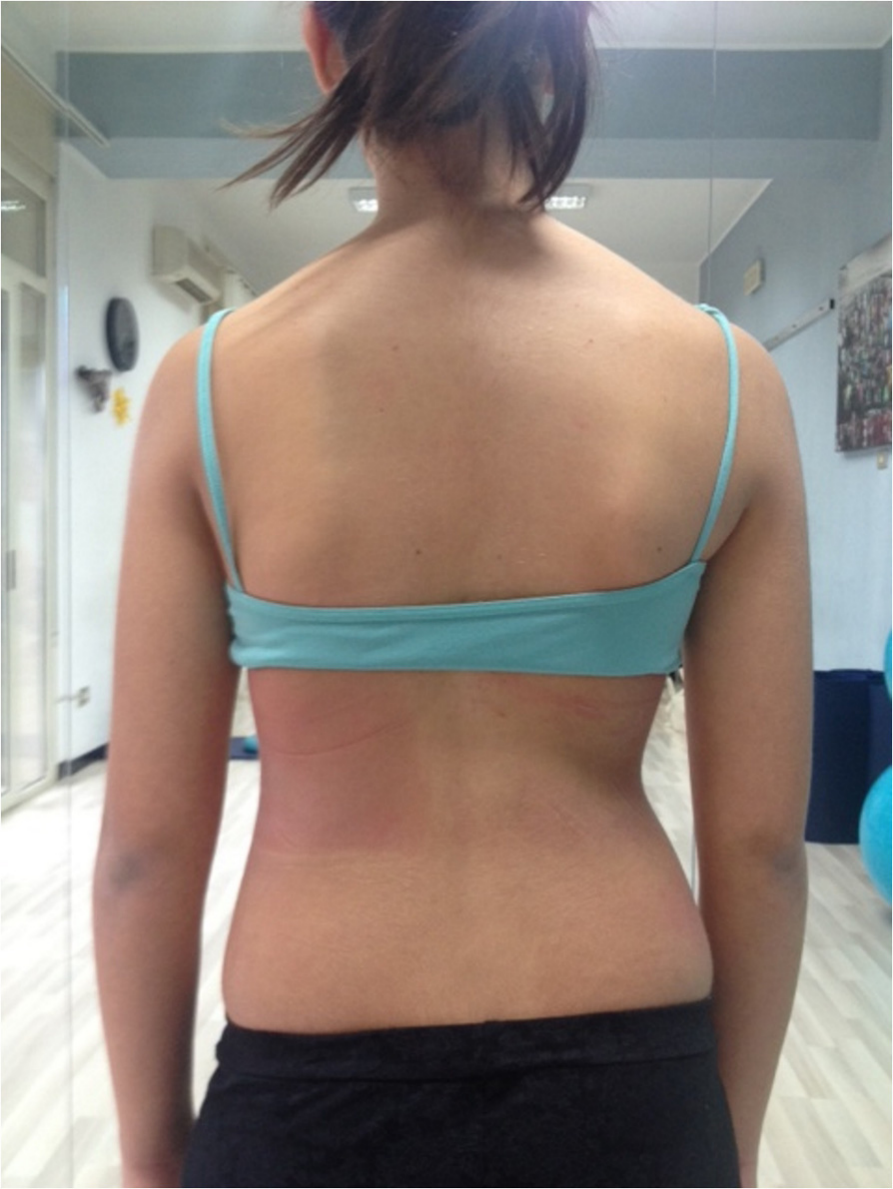
**Patient 1.** Posterior view.

**Figure 2:**
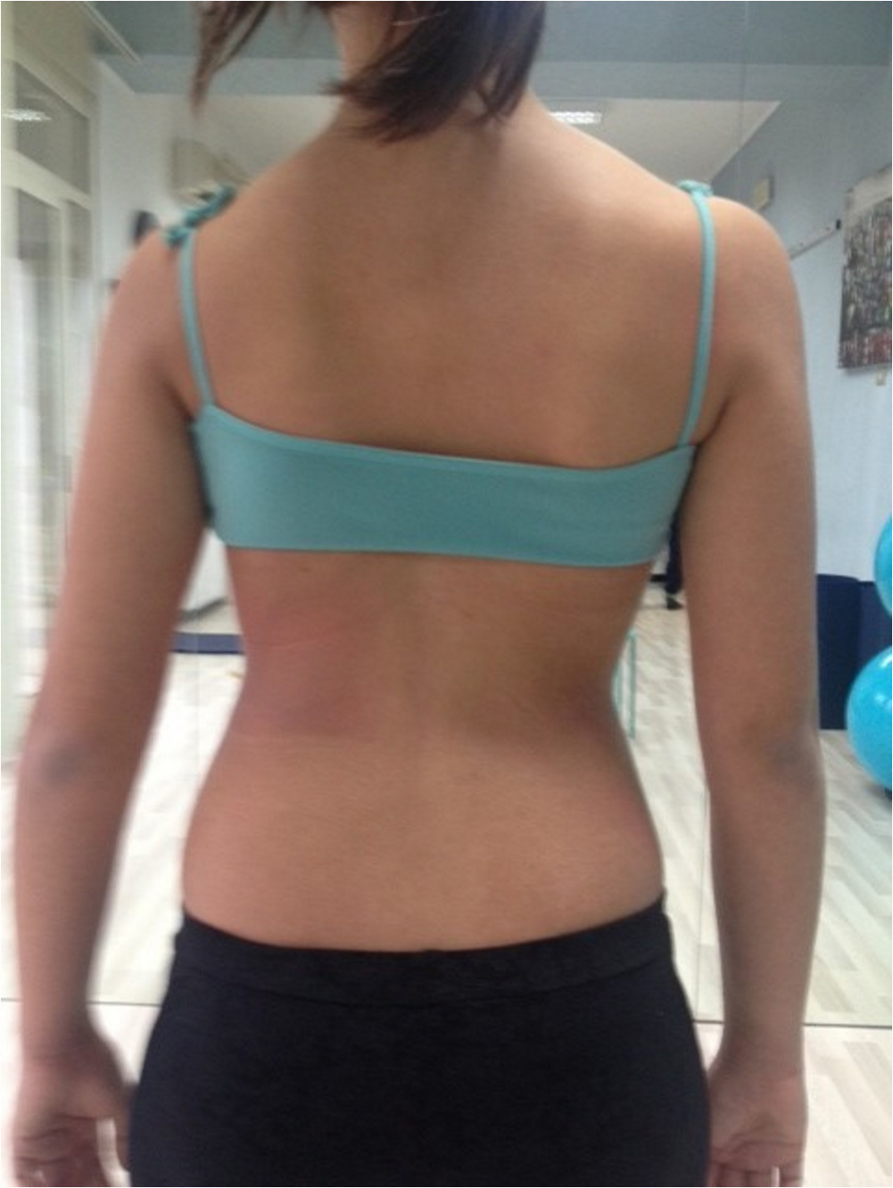
**Patient 1.** Posterior view in active self-correction.

During the execution of “active” self-correction, one can observe (Figure 1 without correction – Figure 2 with correction):

- Significant immediate improvement of the aesthetic of the torso by improved symmetry.
- Improvement of the frontal balance and weight distribution within the spine and through the peripheral joints.
- Improvement of the postural alignment of other body parts (eg. head, elbows).

**Figure 3:**
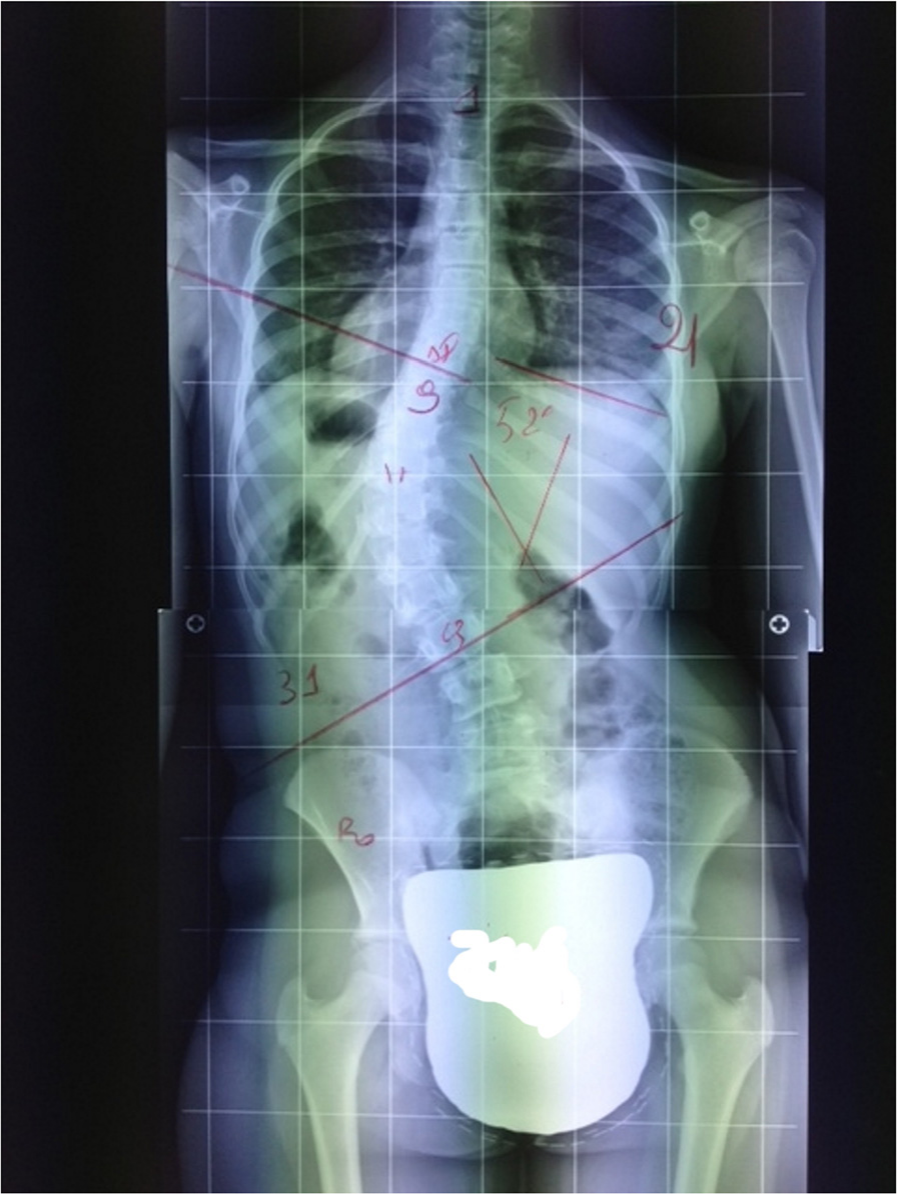
**Patient 1.** Postero-Anterior Spine X-ray.

**Figure 4:**
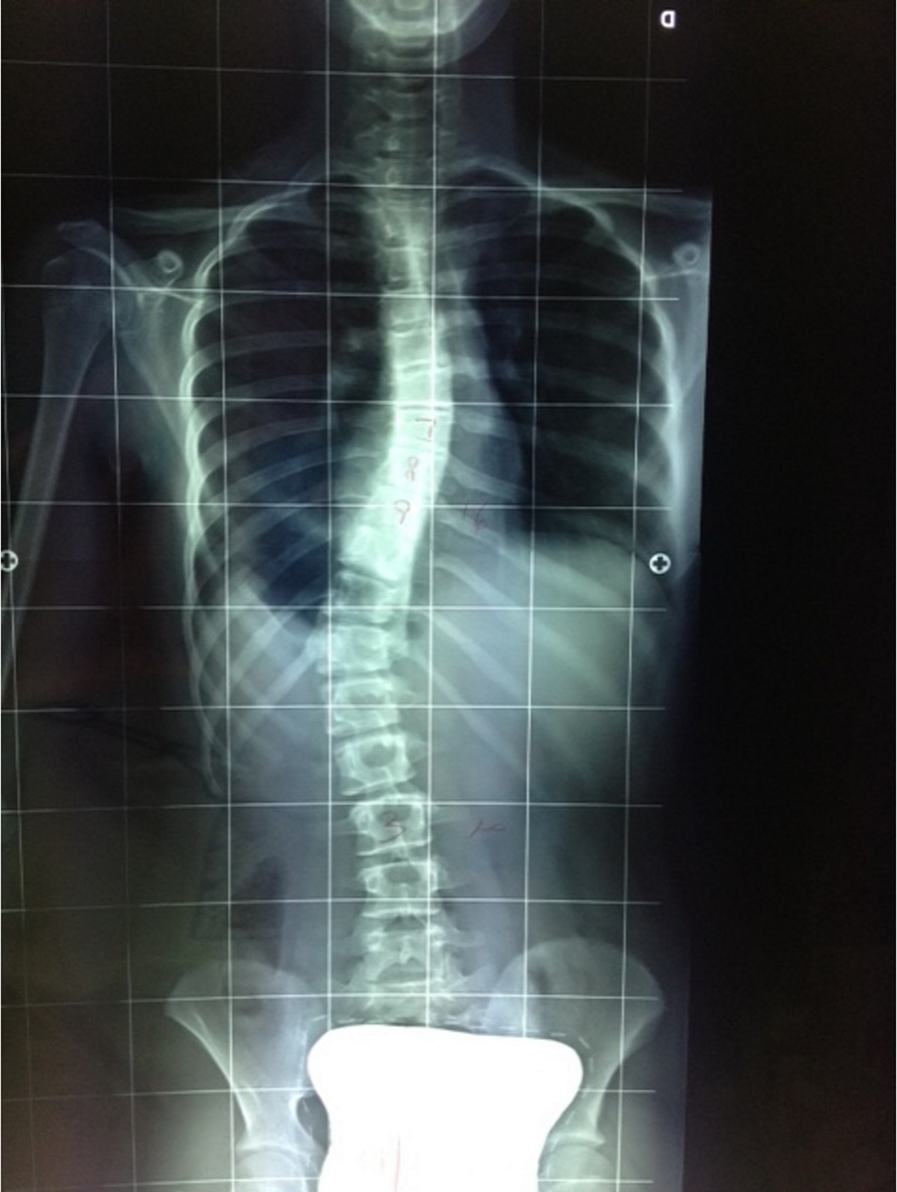
**Patient 1.** Posterior view Postero-Anterior Spine X-ray while the patient maintains the active slf-correction learned during the exercise session.

The changes, however, are not only postural but can also be measured via X-ray (Figure 3 spine without correction - Figure 4 spine while the patient maintains the correction learned during the exercise session).

## Stabilization

Scoliosis conservative treatment aims to counteract progressive vertebral deformation, as determined by the constant disharmonic pressure upon the vertebrae [31,32]. With this purpose in mind, you can use very challenging treatment approaches that require exercise sessions for various hours of the day. However, it is very difficult to maintain the correct position beyond those sessions.

The biggest problem in the treatment of a patient with idiopathic scoliosis is the impossibility of working directly on the cause of the problem, which remains unknown [33,34]. Each type of surgical-, orthotic- or exercise-based treatment must act exclusively upon the effects of the disease and thus minimize them. The most obvious effect of scoliosis on the spine is the deviation of the vertebrae from their normal alignment. Each and every professional who treats scoliosis - the surgeon with surgical implants, the physician and orthopaedic technician with a brace, the physiotherapist with self-correction - seeks to reduce this effect. Because all evolutionary scoliosis tend to collapse down, we can consider the scoliotic spine as an unstable element [35], which the professional seeks to remedy through the use of the tools at his or her disposal. Those with a scalpel take advantage of spinal fusion, while those who use a brace, use the passive support of the spine to provide improved spinal alignment promoting symmetrical growth thereby preventing curve progression until skeletal maturity. Meanwhile, those who use exercises, endeavour to strengthen the stability function in order to counteract curve progression (trend of the evolutive scoliotic spine to move downwards due to the loss of the alignment).

The concept of spinal stability function start from the old Junghans theory of the mobile segment [36], and some biomechanical facts [37]: it is mainly a neuromuscular function [8], based on the action of muscles, but going beyond a simple muscular contraction. In fact, while some muscles like multifidus and transversus abdominis have been shown fundamental for the stability of the spine [7], and while the role of diaphragm and pelvis floor muscles cannot be ignored [38], this function depends on the ability of the central nervous system to coordinate all the different muscle actions [8,39]. It goes beyond the aim of this paper to focus on this concept, but there are plenty of studies in the scientific literature of the last years.

In the SEAS approach, the improvement of spinal stability in active self-correction is the primary objective.

## SEAS exercises

If one uses the term “exercise” to explain the movement a patient performs in order to counteract a disease, in case of scoliosis the exercises specific to its treatment are intended to have a corrective effect on curvature. In this case, as in most scoliosis-specific exercise approaches, self-correction is integrated into such movements. Contrarily, in the context of SEAS, these two elements (self-correction and exercise) are not performed at the same time, but are carried out in succession. Self-correction is the true movement against misalignment. “Exercise” is added to self-correction in order to train the automatic response to maintain an optimal alignment through the widest possible array of challenging activities. Accordingly, exercise is only one element of the complex activities the patient must perform in order to counteract his or her curvature. Exercise is the element of “distraction” that challenges the ability to maintain the self-correction.

In practice, patients perform first the active self-correction, and then the exercises: these have the aims of challenging the obtained correction (so training a reflex answer toward correction), improving stability function and reducing any functional impairment identified in each single patient according to the physiotherapeutic and medical evaluations. These impairments can be patient and/or scoliosis specific (eg balance, eye/hand coordination, muscles retractions, little neurological deficits, etc.), and are identified specifically in each single individual. In this way exercises are scoliosis specific, but also totally personalized to patients’ and treatment needs, continuously changing in time in each individual through gradual refinement and difficulty increase in a real training process.

## The exercises

SEAS exercises have the following two main objectives, in order of importance:

1. The exercises aim to improve the main spinal function, i.e. spinal stability.
2. The exercises aim to improve eventual impairments that the initial evaluation may highlight (strength, muscular retraction, motor coordination, etc.).

### Objective 1: increasing spinal stability

One of the main functions that we want to improve with the treatment, is the stabilization of the spine, namely, the efficacy of the stabilizing muscle of the spine to counteract the evolution of the curve.

The evolution of a progressive scoliosis always runs towards a worsening condition. The scoliotic spine can therefore be explained as a structure whose constituent elements are no longer able to maintain their physiological alignment. The asymmetrical distribution of the load and the progressive deformation of the vertebrae gradually decrease the spinal column’s capacity to maintain stability. Consequently, one of the main objectives of the exercises provided in this approach is the stimulation of the muscles with the highest potential for stabilization (i.e. maintaining the spine’s proper alignment). The purpose is achieved by asking the patient to perform the actions that challenge the stability of the spine such as balance exercises, exercises using an additional load, exercises that include dynamic components and other challenges related to the deficits detected during the initial assessment.

### Objective 2: improving function

The SEAS approach provides a thorough evaluation of the patient before the treatment starts. In addition to the usual measurements for the specific assessment of the scoliosis (Cobb degrees, Bunnel, trunk decompensation, sagittal postural structure and aesthetic parameters), the patient performs a series of tests. The objectives are:

- assessment of physical conditions (strength and elasticity of certain muscle areas that are more likely to influence the structure of the pelvis and the spine).
- assessment of neuromotor ability related to the alignment of the spine and the pelvis (balance, management of body movements with closed eyes, optical/manual skills).

The purpose is to obtain a reliable general diagnosis of the patient. The information is used to guide the selection of the exercises to improve any impairment identified during the course of the evaluation. Some of these data are also useful to be identified, as there are different types of exercises that are most convenient for the improvement of the spinal stability. For example, if during the course of the evaluation clear signs of balance difficulties are found, those exercises which have the corresponding balance elements will be used to improve the stabilization capacity of the spine. The assessment takes about 20 minutes and it is performed on a regular basis: the tests having shown any specific impairment are usually performed every three months, while the complete evaluation is usually done every year.

## Active self-correction

### Learning self-correction

**Figure 5:**
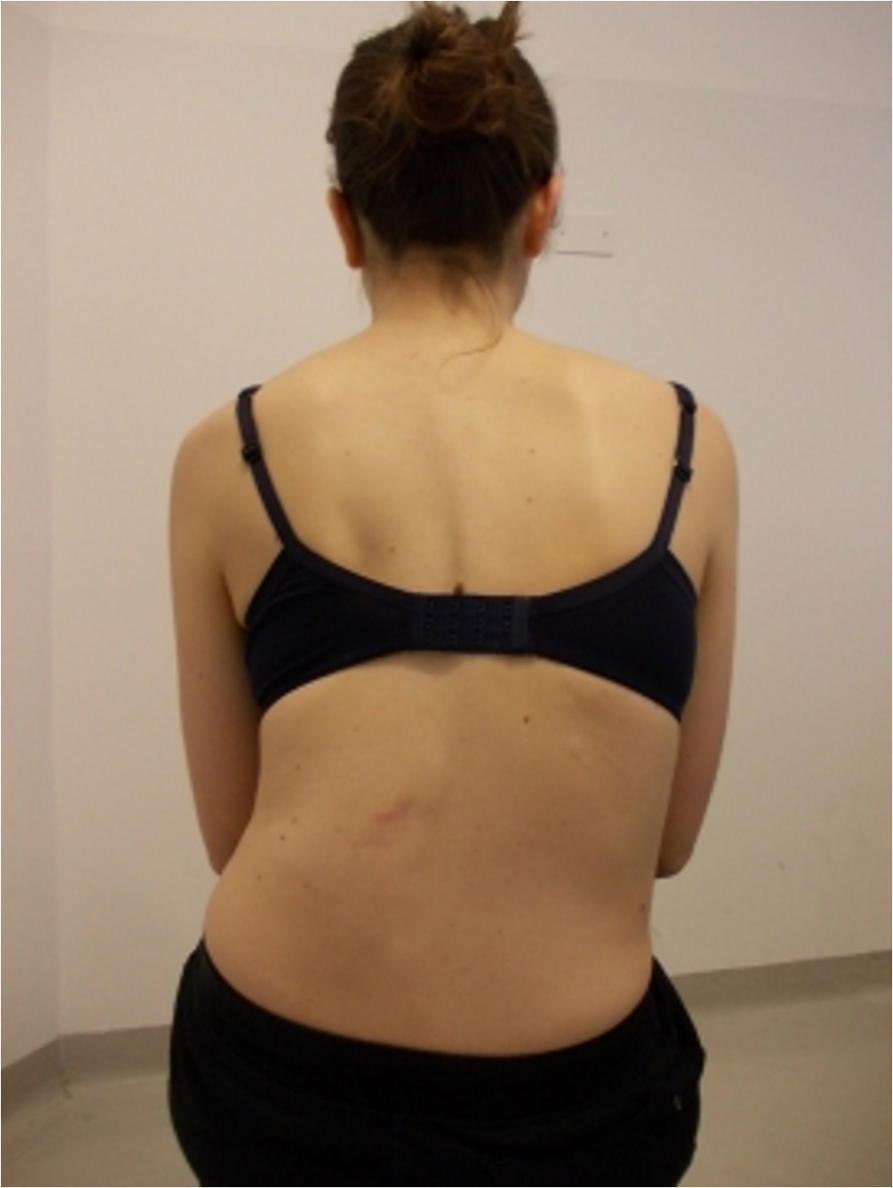
**Patient 2.** Sitting position: start of exercise. Patient in uncorrected relaxed posture.

**Figure 6:**
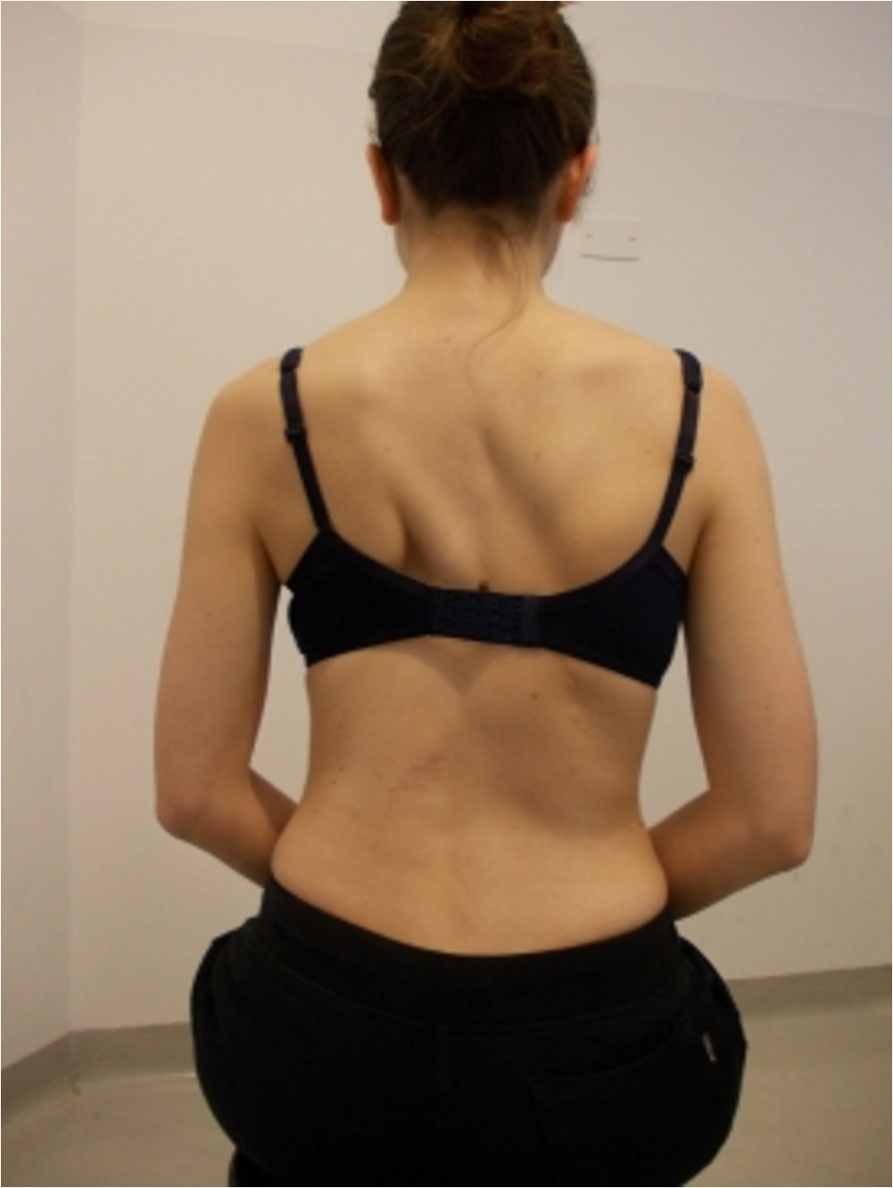
**Patient 2.** Active self-correction in sitting position.

**Figure 7:**
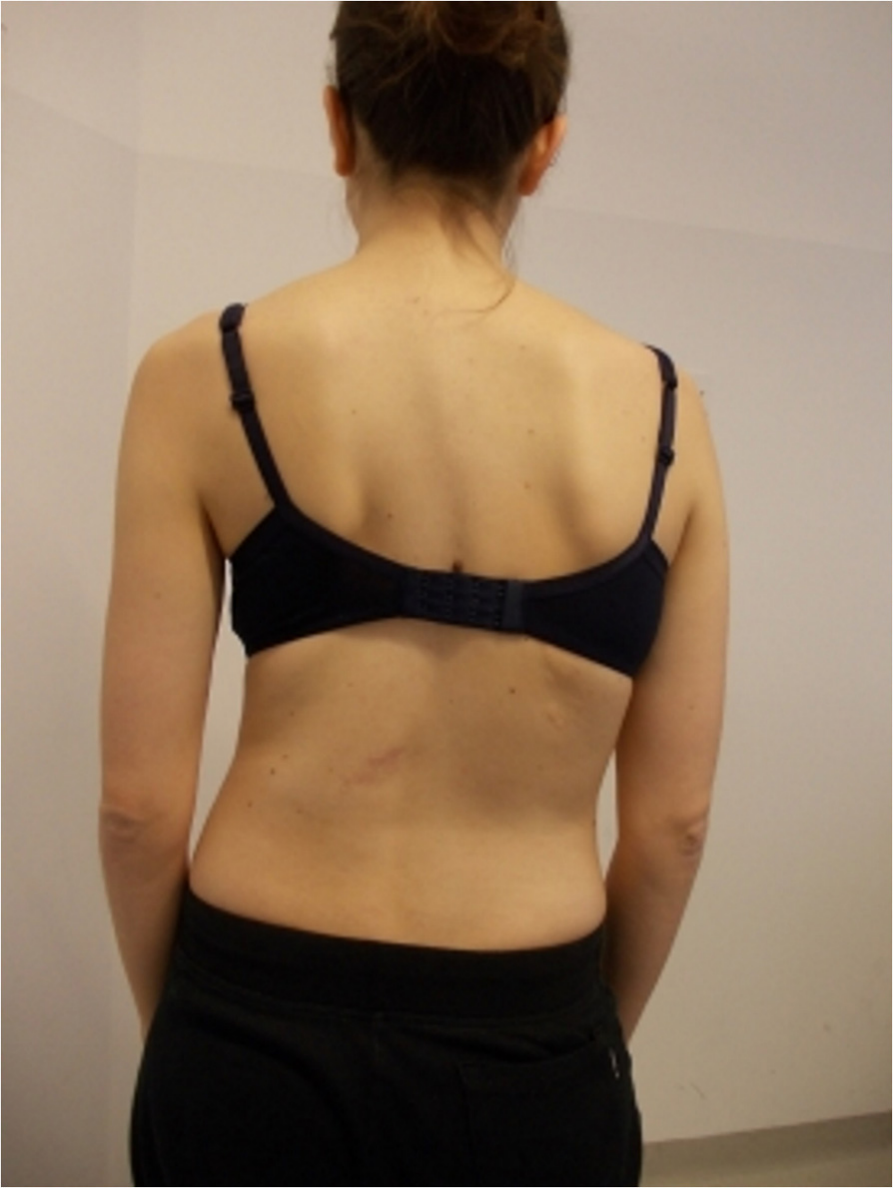
**Patient 2.** Standing position: start of exercise, Patient in uncorrected relaxed posture.

**Figure 8:**
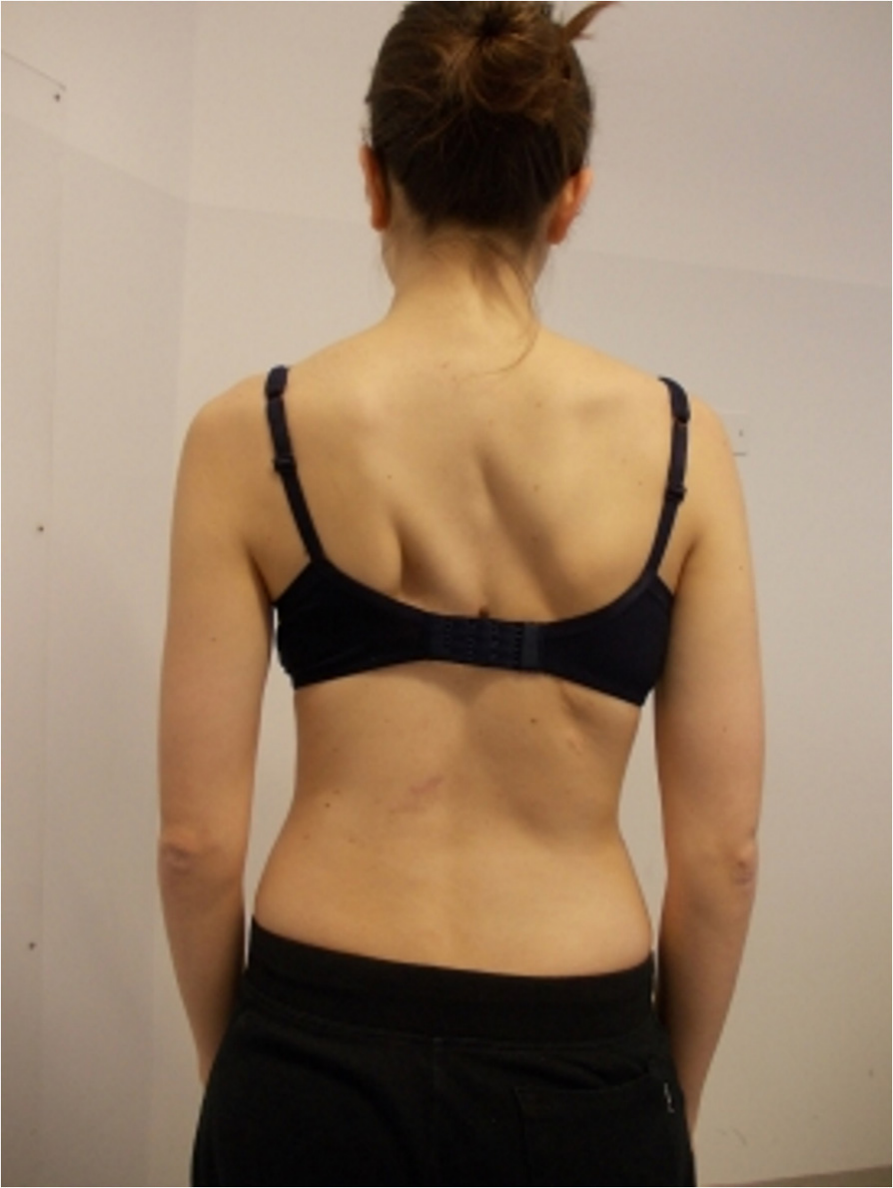
Patient 2. Active self-correction in standing position.

Active self-correction is a corrective movement selected according to the patient’s impairment, the morphologic features and posture. The aim of self-correction is to restore a position as close to physiologically normal as possible. Self-correction is composed of movements performed in all spatial planes – coronal, sagittal, horizontal – in an overall vertical anti-gravitational direction. The patient learns how to perform the corrective movement in different positions (in a standing or in a sitting position, on all four…), (Figures 5, 6, 7 and 8) because the exercises are performed in a manner that simulates situations and movements in daily life.

- The movement in the coronal plane is named translation and aims to reduce the curvature’s Cobb angles. The word “translation” means the coronal displacement of the apical vertebra towards the mid-line. It is always performed obliquely upwards, to counteract the postural collapse (with this concept we want to emphasize that the tendency for the evolutive scoliotic spine to lose height as it bends and twists over time). Like for SPoRT bracing [22], the active self-correction focuses on the vertebra that shows the most serious coronal tilt.

The movements in the sagittal plane are flexion (usually in the thoracic spine: kyphotization) and extension (usually in the lumbar spine: lordotisation). Their aim is to recover physiological sagittal curves. The choice of the correct sagittal movement obviously depends on the actual situation of the individual patient, since the thoracic spine does not always have to see the kyphosis increased and the lumbar lordosis does not always require an increase.

- The movement in the horizontal plane is named derotation and aims to reduce spine torsion.
- The vertically directed movement is named elongation.

To simplify the learning of active self-correction, follow these indications to facilitate the performance by the patients and reduce the decompensation:

- Before starting active self-correction, the patient stays active, to recover his/her relaxed posture.
- The correction begins preferably in the more caudal area, i.e. first the patient corrects the lumbar spine and then the thoracic spine.
- When a more cranial section is corrected, the movement should not provoke the loss of control of the lowest correction.
- At the lumbar and thoraco-lumbar level of the spine, the correction of the sagittal plane precedes the correction of the coronal and/or horizontal plane.
- At the thoracic level, the correction of the coronal or horizontal plane precedes the one performed on the sagittal plane.
- The correction of the coronal or sagittal deviation of C7 relative to S1 is integrated into the self-correction movement.
- In the case of a double curvature, the correction of the primary curve preferentially precedes the correction of the compensatory curve.
- In the case of a double curvature, where it is difficult to determine the primary curve (importance of Cobb degrees, height and characteristics of the humps, rigidity of curvature), the correction of the caudal curve precedes the cranial curve.

During the learning stage the patient can help himself with various resources, particularly a mirror. The help of a reference point is crucial at this stage. It is true that a visual control does not facilitate the more specific neurological fibers providing sensory information about the trunk position in space [8], because these fibers aren’t linked to sight, but to the perception of the body in space. However, during the initial period it is difficult for the patient to learn and replicate complex movements and targets, such as those of self-correction, without using visual feedback. The mirror therefore represents an essential tool during the initial phase, but it should be eliminated gradually in order to train the patient in the management of the trunk position under the control of the most specific proprioceptive fibers in this region, namely the somatosensory context. The usage of the mirror, that was used since the beginning also by the Lyon School, can also have a very important neurophysiological effect through the activation of the mirror neurons [40,41].

The breathing mechanism of the patient is another fundamental advantage. The patient can more easily perform any movement that requires great attention and concentration by helping himself with breathing. The danger is that he/she could get used to maintaining the correct position with breath held over the duration of the exercise. One of the first things the patient must learn to do is to empty the lungs and breathe normally while keeping an active self-correction through the exercise. In fact, one of the first exercises that may be required for the patient is to breathe quietly in a self- corrected position.

Generally, the maintenance of a self- corrected position at the beginning is very tiring. It is recommended to explicitly refer to the tiredness felt by the patient while trying to maintain the position. If the patient responds that he/she does not feel tired, it is probably because he/she is not performing the exercise properly.

## The four basic questions for self correction during exercises

If it were necessary to define in a single word the fundamental principle on which the SEAS approach is based, that word would be “control.”

The patient is encouraged to verify in each moment the performance of the proposed exercise and that the self-corrected posture is being maintained. To facilitate this control, the patient answers a series of standard questions that arise during the performance of each exercise.

In the classical SEAS approach, the role of the physiotherapist is to teach carefully the active self-correction and the exercises, so as to allow the patient to be able to perform the treatment by himself. Consequently, the following questions are for the patients, to be applied in their everyday performance of the exercises.

This approach gives autonomy, and in different settings allows the patients to perform carefully the exercises in little groups, with only one supervisor (trainer). If the physiotherapist follows one patient directly, these questions should in any case be asked to make him try to achieve the best possible correction with the maximum of active participation.

### First question: relax

One of the items required from the patient who performs the exercises, is to start always from a position where the spine has an adequate base of support. This means that the patient should confirm, before performing each exercise, that he/she is not in a relaxed position. Thus the first question to be **posed is: Am I relaxed or active? At this sta**ge of the exercise, the patient does not yet perform the correction. Regardless of the patient’s starting position (sitting, standing), the only requirements are to have a base control of the spine and not to be in a relaxed position. Once this precondition is verified, the patient adopts the self-corrected posture.

### Second question: symmetry

As a means of verifying the correct performance of the self- corrected posture, the patient poses a second question: **Is my trunk more symmetrical than before?** The verification is initially visual (“Can I see that my trunk is more symmetrical than before?”), because the patient is performing the exercises in front of the mirror. Eventually, however, he/she will be more influenced by the somatosensory perceptions (“Can I feel that my trunk is more symmetrical than before?”), because later the exercises will be performed without the assistance of the mirror.

### Third question: challenge

The patient now performs the exercise that will have the purpose of making it more difficult for him to retain the self- corrected posture. The question he/she will raise at this point is: **The exercise is challenging my ability to maintain the self-correction, am I able to maintain the self-corrected posture?** Based on the patient’s reply, the therapist can understand whether the level of difficulty of the exercise is appropriate to the patient’s ability to maintain the self- corrected posture during such an exercise. In fact, if the patient replies “no” to this question the therapist should have the patient perform an exercise that poses less difficulty.

### Fourth question: return

The patient performs the exercise for approximately ten seconds and then slowly relaxes the self- corrected posture. The question he/she asks now is: **Can I still notice a difference between my self-corrected position at the end of the exercise and my natural position?** The patient should be able to see that his trunk position goes from the self- corrected posture to the usual one. This is probably the most important verification the patient performs in order to understand whether he/she has correctly performed the exercise. The relaxation stage is the one where the shifting of body mass occurs through elastic retraction without following an active movements. If the patient answers “no” to this question, it means that, during the performance of the exercise, the self- corrected posture had been lost and the performed challenging exercise had led to the loss of the corrected characteristics and had become a simple gymnastic exercise.

Clearly, this training strategy aims to improve the awareness of the patient to correctly perform the exercises.

The ultimate goal of the treatment is to train the patient to maintain the longest possible time the self correction throughout daily life to truly fight Stokes vicious circle.

## Examples of the sequence of actions for two SEAS exercises

### Example of a basic exercise

For this patient it has been chosen to perform the self-correction in the coronal plane, since he/she is not able at this stage to integrate a true and complete three-dimensional self correction. The task required the patient to move from the sitting position to the standing position while preserving the self- corrected posture throughout the exercise. The patient should carry out the exercise in ten to twelve seconds.

**Figure 9:**
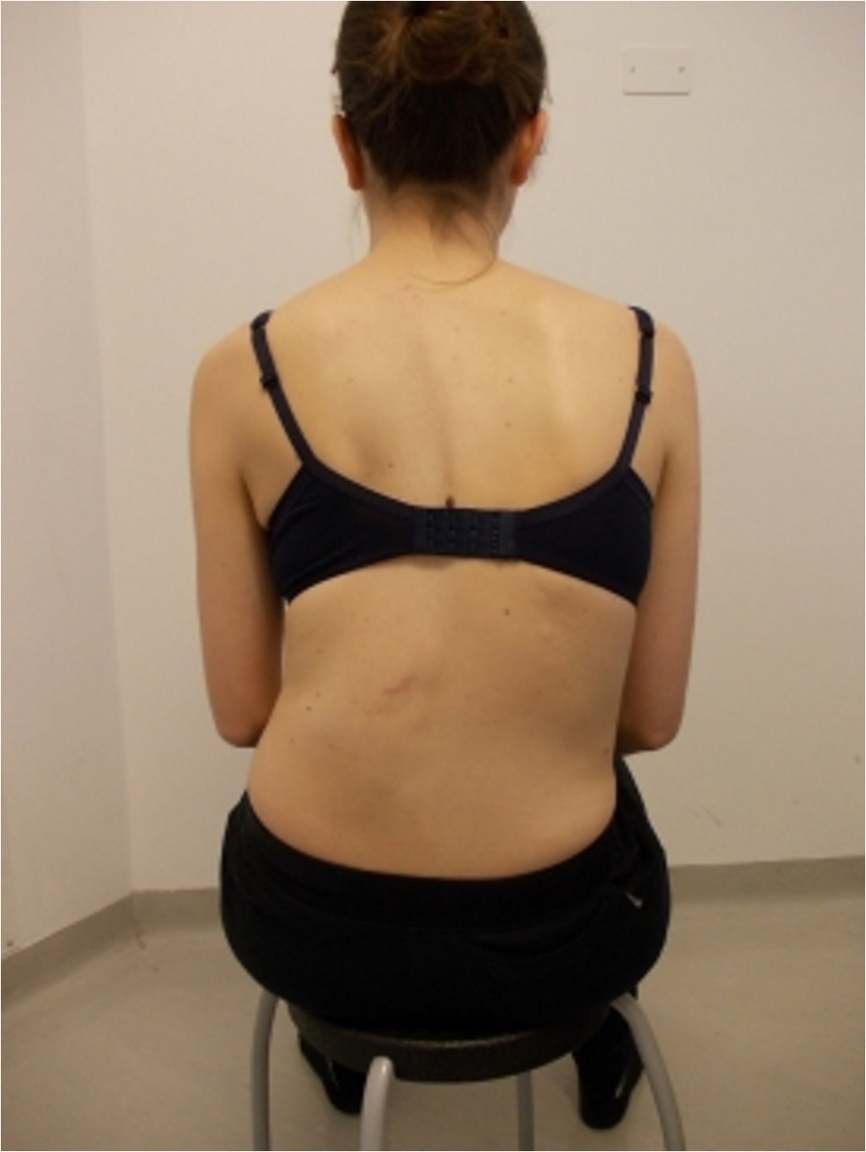
**Example Exercise 1.** Patient in sitting and uncorrected relaxed posture.

**Figure 10:**
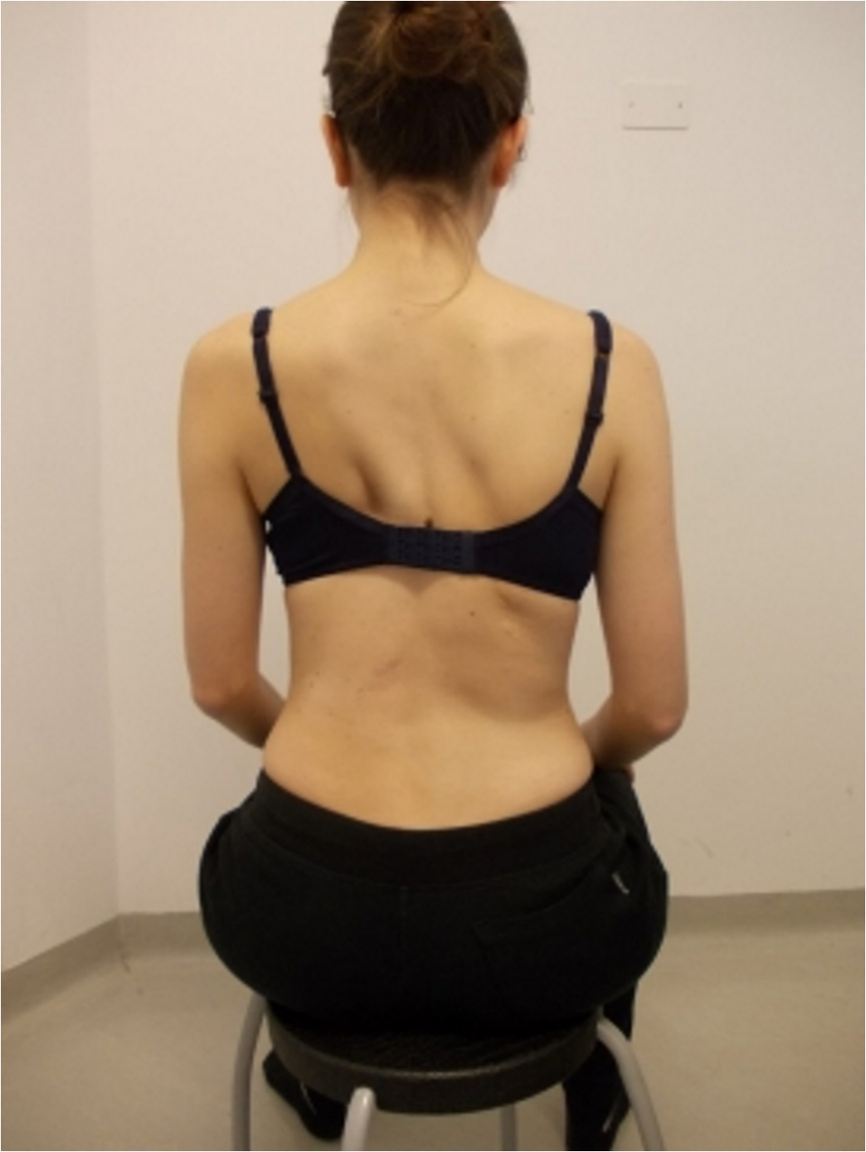
**Example Exercise 1.** The patient perform the active self-correction.

**Figure 11:**
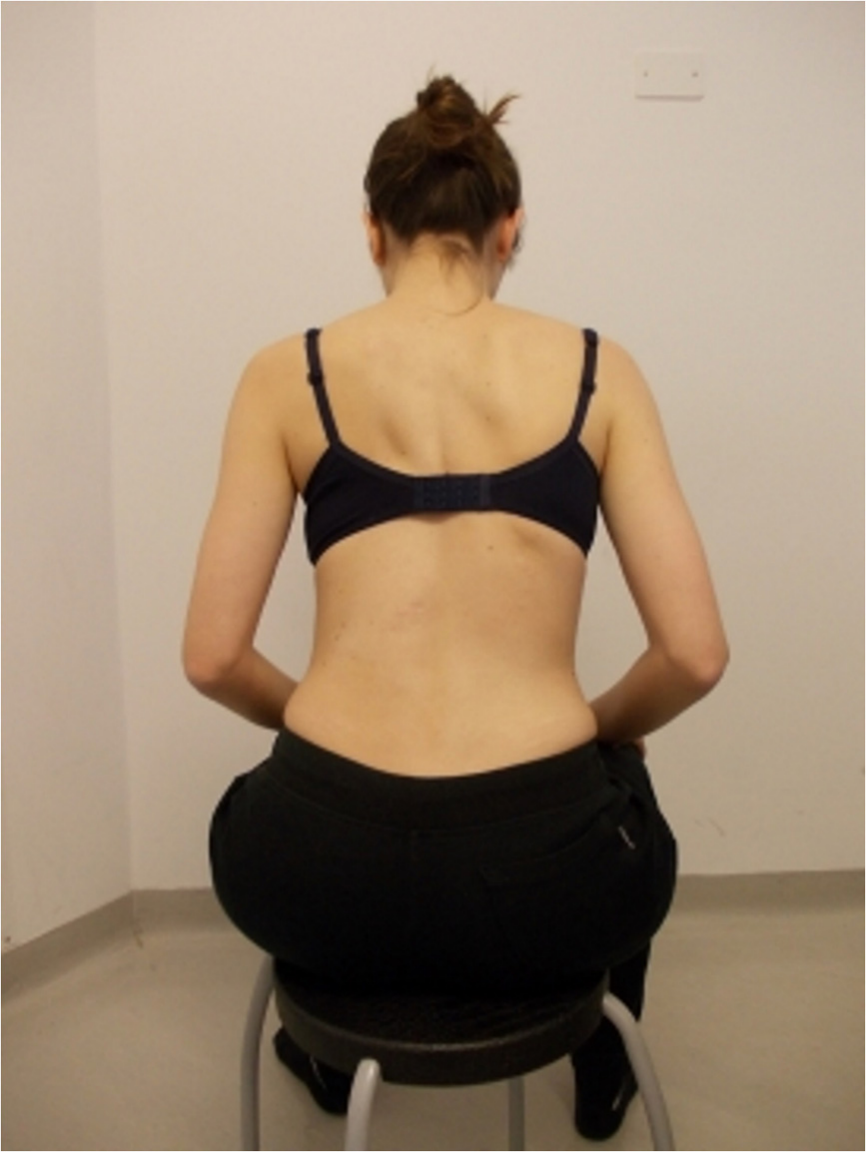
**Example Exercise 1.** The patient lean forward preserving the physiological sagittal curves and the active self-correction.

**Figure 12:**
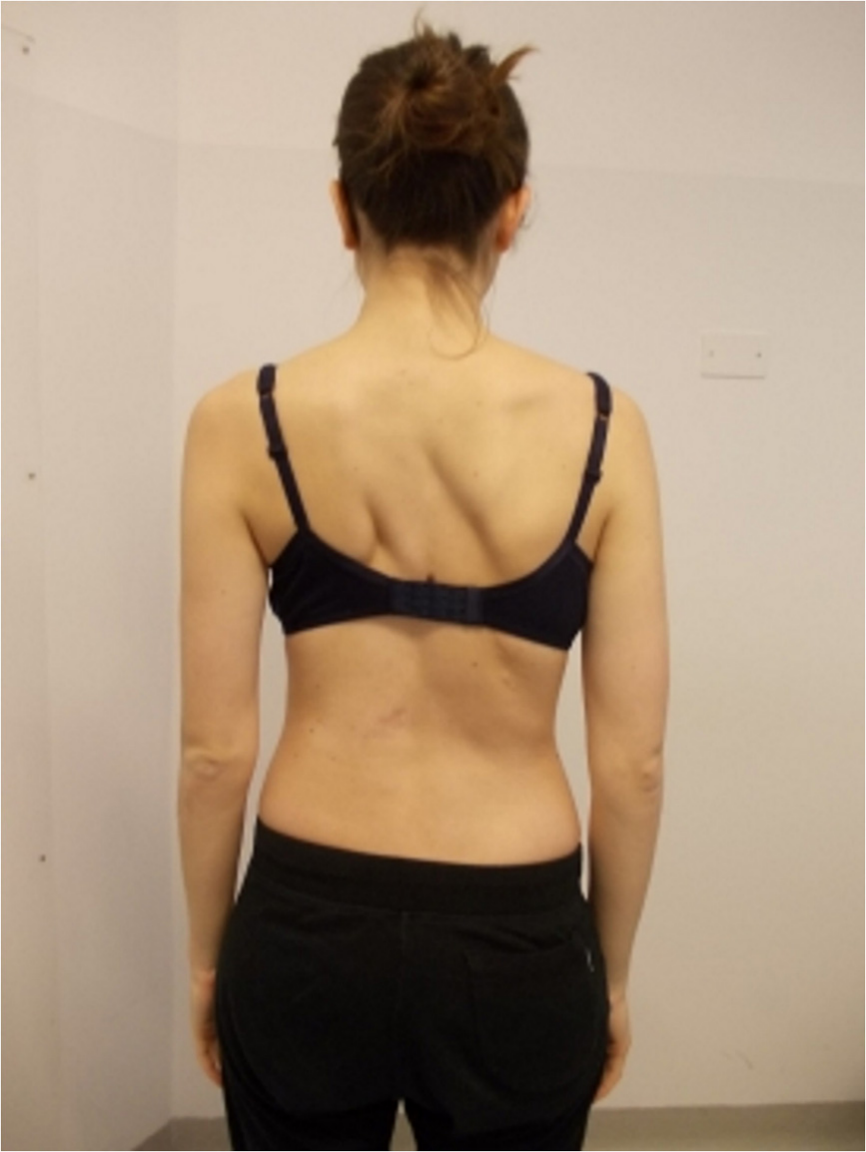
**Example Exercise 1.** The patient reaches the standing position and maintains the active self-correction.

**Figure 13:**
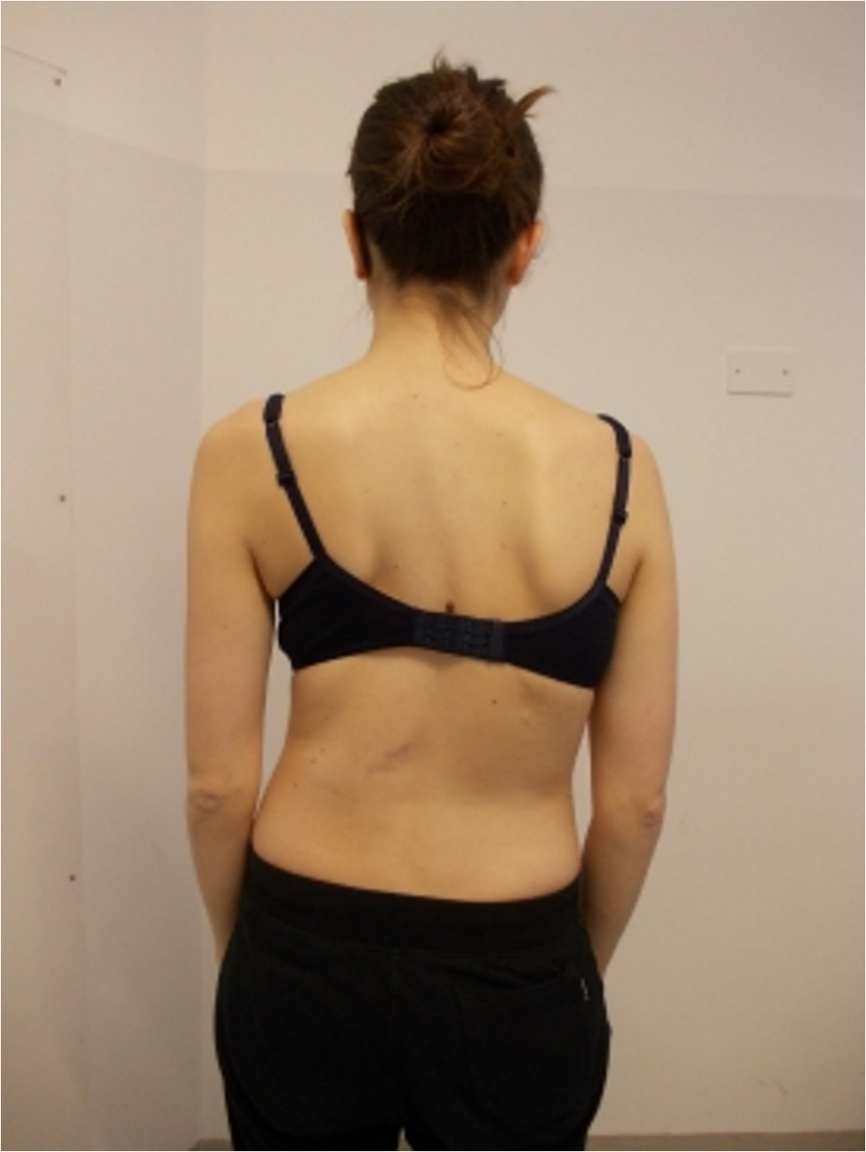
**Example Exercise 1.** The patient relaxes from the active self-correction.

The patient was in a seated position (Figure 9) and started to perform the correction of the right thoraco-lumbar curve with a lateral translation towards the upper part of the thoraco-lumbar section towards the concave side (from the right to the left) (Figure 10). The patient leaned forward while preserving the physiological sagittal curves and the correction on the coronal plane (Figure 11). The patient reached the standing position and maintained the corrected posture (Figure 12). The patient relaxed from the self- corrected position (Figure 13).

### Example of an advanced exercise

The task requested to the patient consists of falling towards a wall, landing on the hands, bending the elbow and pushing in order to return to the starting position while keeping the self- corrected position during the exercise. The patient should perform the exercise for approximately ten seconds for each cycle from starting position to returning to the starting position. For this exemple it has been chosen to perform the self-correction on the horizontal and sagittal planes, since this is enough for the patient to achieve the best correction.

**Figure 14:**
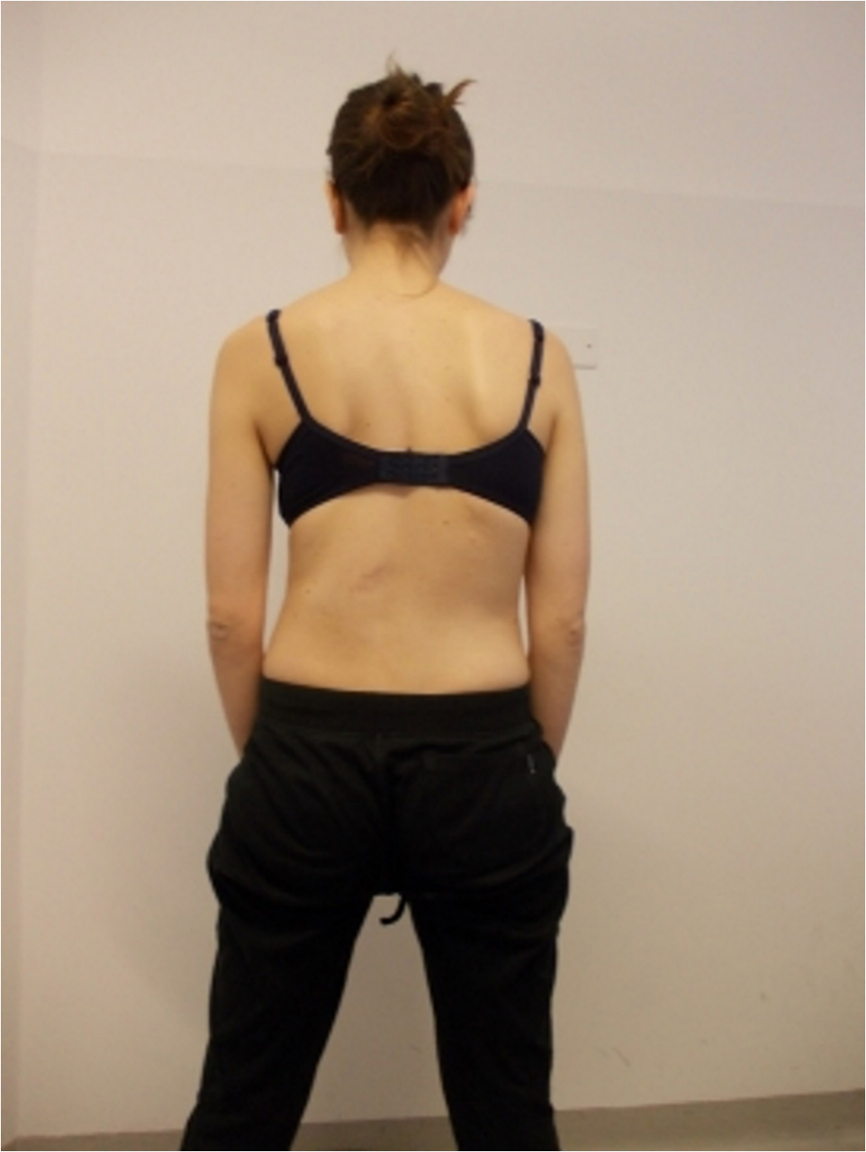
**Example Exercise 2.** The patient in standing position in front of a wall. The patient is in uncorrected relaxed posture.

**Figure 15:**
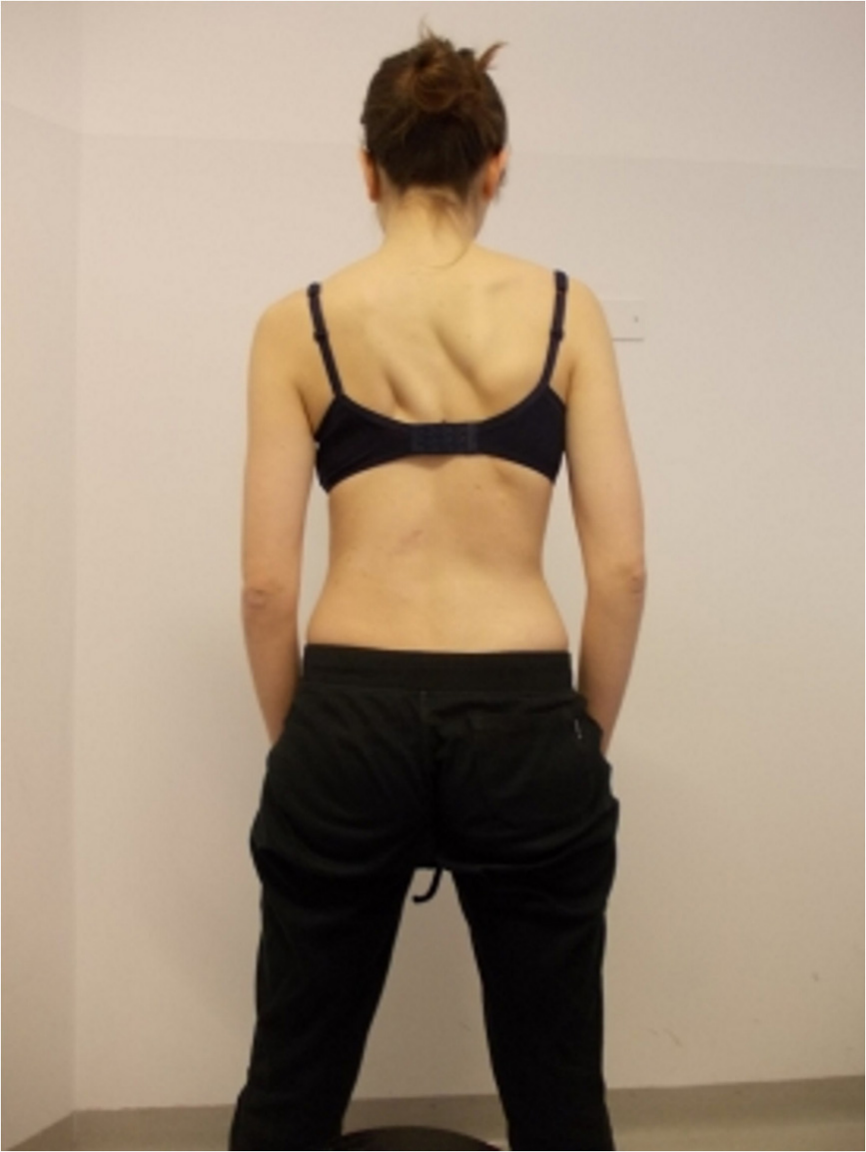
**Example Exercise 2.** The patient performs the active self-correction.

**Figure 16:**
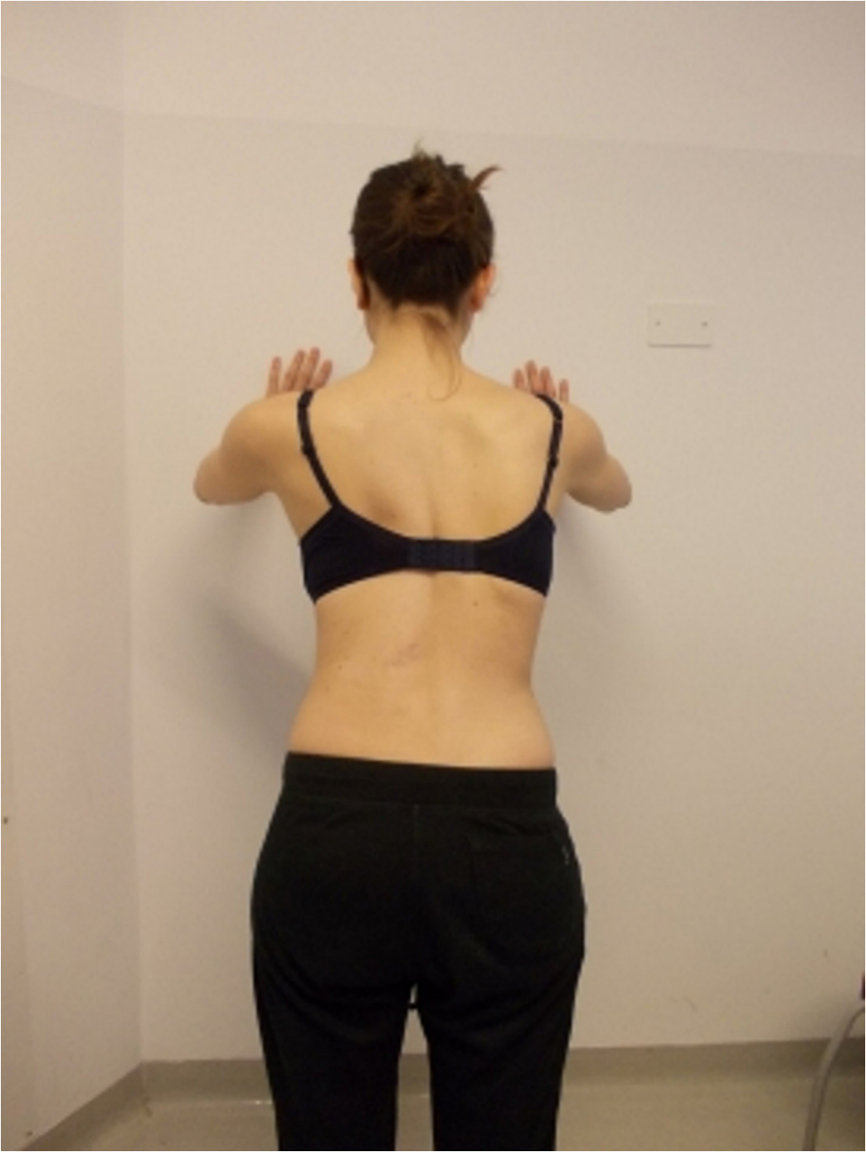
**Example Exercise 2.** The patient falls against the wall, landing on both hands and keeping the active self-correction.

**Figure 17:**
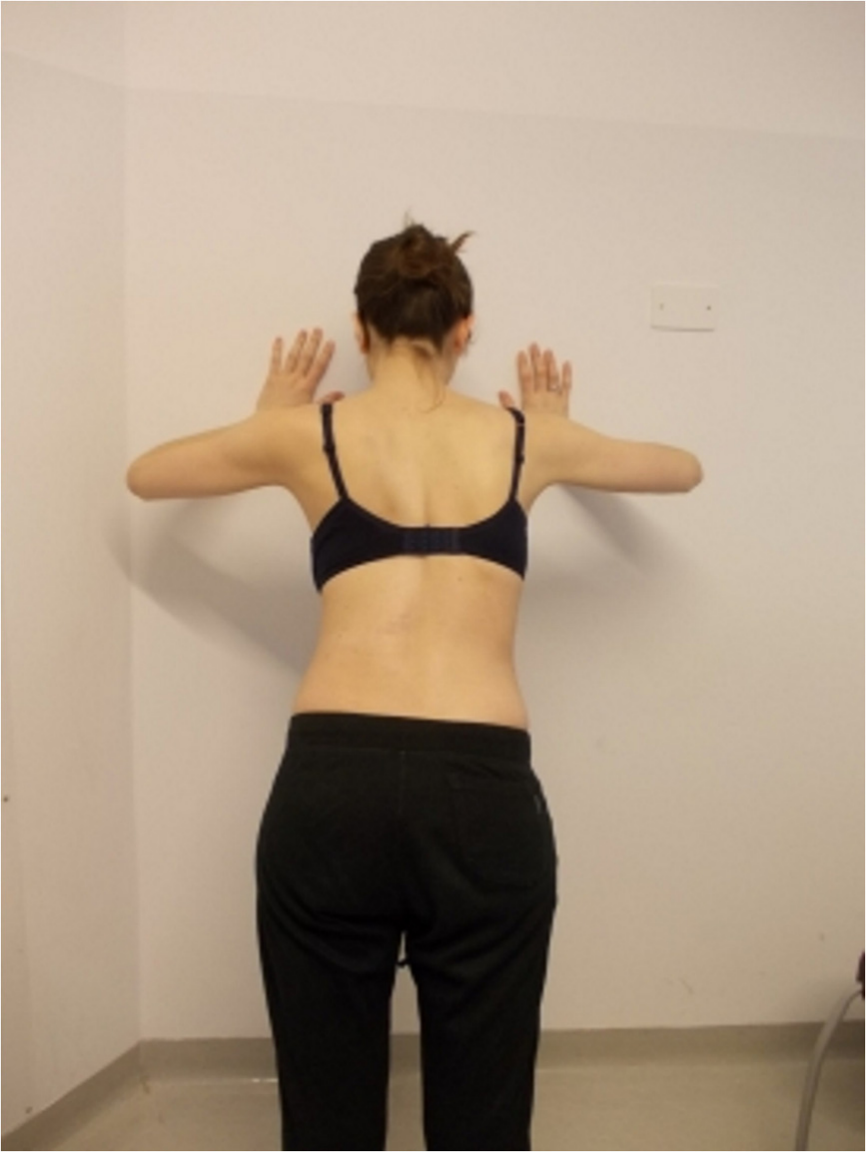
**Example Exercise 2.** The patient bends the elbows and then pushes back with the arms keeping the active self-correction.

**Figure 18:**
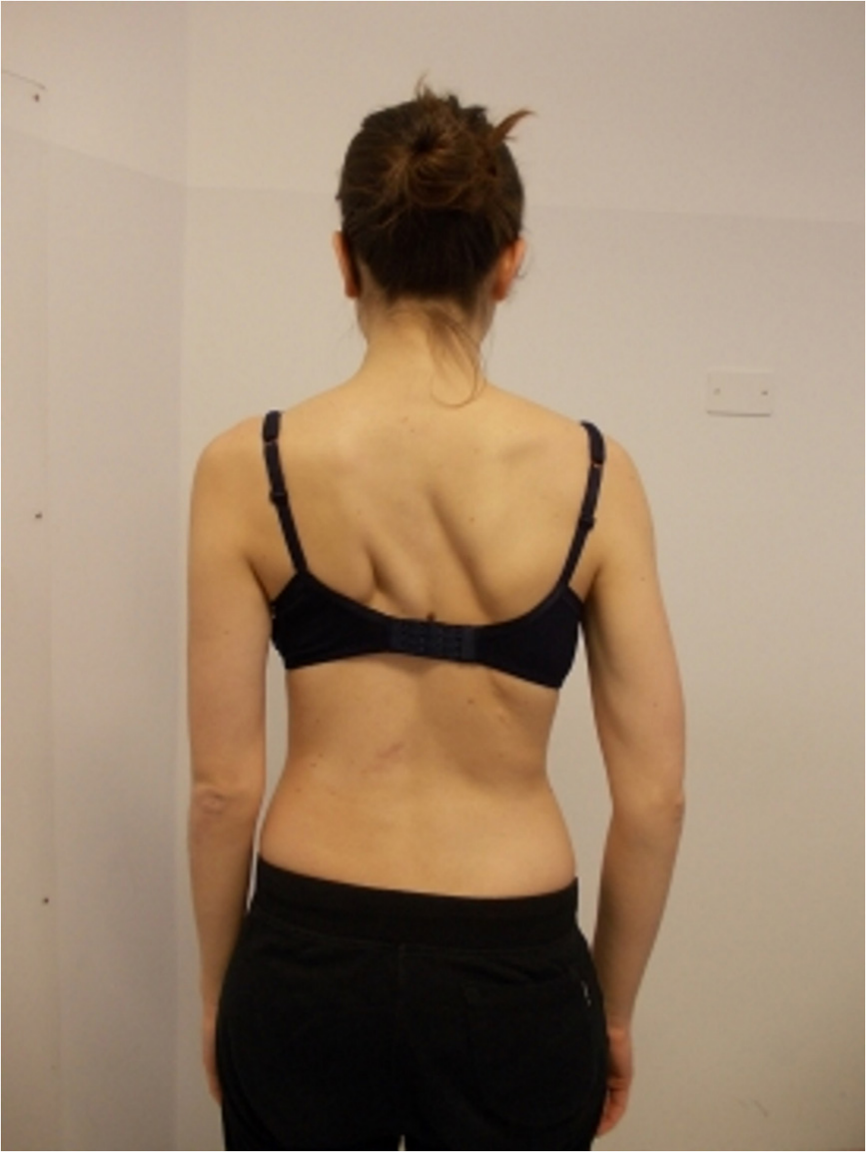
**Example Exercise 2.** The patient return to the starting position without losing the active self-correction.

**Figure 19:**
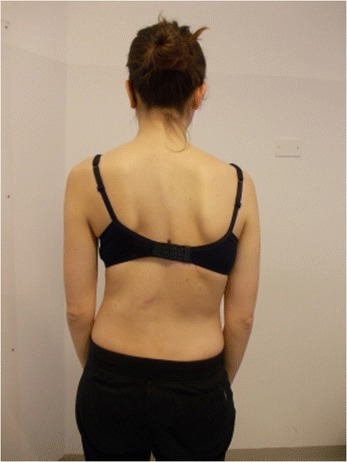
**Example Exercise 2.** The patient relaxes, losing the active self-correction.

The patient is standing in front of a wall at approximately 80 cm distance (Figure 14). In this example the patient performs only the thoracic self-correction in the horizontal plane with a counterclockwise rotation, and stabilizes the lumbar spine as much as he/she can (Figure 15). The patient falls against the wall, landing >on both hands and keeping the self-correction (Figure 16). The patient bends the elbows and then pushes back with the arms (Figure 17) in order to return to the starting position without losing the self- corrected posture (Figure 18). The patient relaxes, losing the self-corrected posture (Figure 19).

## Practical issues

### Protocols for the application of the SEAS exercises

The keywords for SEAS application include:

- Control, as stated above with the four questions
- Learning: the entire process is a neuro-motor improvement based on new motor experiences; accordingly, difficulties must be increased and feedback reduced, with learning.
- Training: repetition is the basis for learning;
- Individualization: both active self-correction and exercises are totally individualized according to patients’ physical abilities and needs;
- Self-care and autonomy: patients learn through involvement and individual application; this is true in all protocols, in either outpatient or home-program regimens: in the first one patients never performed the same exercises all together, in the second independency and self-care are mandatory;
- Cognitive behavioural approach: SEAS exercise learning includes furthermore the time for a regular family counseling at the end of each session

Many different protocols can be applied. In the past, the SEAS exercises were applied only as an outpatient group approach, with two/three sessions per week performed by an expert physical therapist. Every two-three months a specific evaluation was performed together with a counselling session. Today, this protocol has improved in some specific clinical situations to a more cost-effective home program. Nevertheless, it is still possible to find in Italy, where the SEAS program is well established among physiotherapists and rehabilitation professionals since many years, the classical outpatient application [42]; sometimes, this unfortunately does not include the individual regular evaluation and counselling sessions.

The typical actual expert application of the SEAS protocol starts with the evaluation of the patient, followed by individual teaching of the exercise program, and ends with a family counseling session based on a cognitive-behavioural approach, to obtain the better compliance of the patient. The SEAS approach attributes a fundamental relevance to this counseling session, because it considers the patient’s family to be a highly important representative member of the therapeutic team. Family support is mandatory to obtain the necessary compliance to achieve the optimum final result.

The modern SEAS approach requires a session that lasts about 1.5 hours. Usually, each session takes place every three months (so, there are four - five sessions a year) and it is carried out by an expert physical therapist. Exercises are recorded on a USB memory media while the patient is learning them, to help the patient remember every detail and repeat the exercises properly either at home with the help of a family member or at a nearby gym, with the assistance of another physiotherapist or personal trainer. If the patient comes from very far away (more than one hour by plane), or in specific situations, it is also possible to have two sessions with the physical therapist every six months thus preparing two treatment plans for the following six months. The patient will start the second exercise session (after three months), usually after a phone contact or a short Internet tele-medicine evaluation.

In either the outpatient or the home program regimen, the patient repeats his exercise program with two or three 45-minute sessions per week or with a daily session of 20 minutes, according to the preferences of the patient and his family. In outpatient settings it is possible to perform the exercises in little groups with a single trainer following up with six to eight patients (usually four to five). In this situation, the exercises are all changed, with one individual session every three to four months.

The progression of the exercise difficulty is based on the improvement of the ability of the patient to keep the correction. The modification of the exercises is the tool for the progressively more difficult *training to* achieve the ultimate target of the treatment: to be able to maintain the correction throughout daily life”..

### Characteristics of the SEAS exercises

The exercises aren’t strictly defined on the basis of a curve pattern. Unlike other therapeutic approaches, there are no specific exercises for lumbar or thoracic scoliosis. This means that the specificity of the exercise does not depend on its use in a given situation but on its proper integration with the self-correction exercise.

In order to train the patient to reach the correct position of vertebral alignment and maintain it for a sufficient period of time, the level of difficulty created by the exercise must be measured carefully. The variables to be considered are the intensity of the challenge (how many seconds the patient must hold the position, how much overload should be imposed, how much the patient should lean over, etc.) and the typology of the postural challenge, namely imbalance, coordination difficulties or muscular effort etc.

For example, if the patient should manifest difficulty with respect to single leg balance or hand-to-eye coordination, the option of introducing exercises that challenge those functions would have a dual purpose: not only to improve the deficit, but also to improve the specific ability of maintaining self-correction.

The exercises are chosen on the basis of the results of the evaluation and in consideration of the patient’s characteristics, exploiting the weaknesses of his functional capabilities in order to adequately train the reinforcement of the stability of the spine.

To explain it better: the muscle response that the corrective exercises should elicit is of a tonic type, that is, the ability to maintain over time a muscle contraction. This is important because trained and strengthened muscles support the spine [29]. Thus the exercises are mainly designed to provide and influence the ability of control. This does not imply that they should be static but that the exercises should be characterized by an optimal control of the alignment of the spine, which in turn is requested by the self- corrected posture.

The exercises should be able to allow an experimentation of very different positions. Daily life requires a wide range of postural combinations, consequently with these exercises we aim to train the reflexive response in self-correction posture, using postural challenges determined by the patient’s needs in daily life.

We need not emphasize the importance of varying one’s practice during the training. We have to plan the inclusion of increasingly more complex dynamic components. Everyday life is made up of actions, so, after a natural learning period that is facilitated by simple and generally static positions, the self-corrected posture should be exercised during the course of actions that simulates the difficulty of real life.

It is necessary to gradually abandon the visual aid as a control channel. Of the three neurological afferent channels concerning the position of the spine in space (visual, vestibular, somatosensory), sight is not the privileged one. This is because a portion of the body is hidden from sight and located in the central part of the spine The somatosensory system is the channel most responsible for the perception of the body in space [30]. For this reason, and as soon as possible, one must encourage the patient to perform these exercises without the support of the mirror.

### Individualization of exercises

One of the distinguishing elements of this approach, as compared to other treatment methods, is the absolute attention to the patient’s individual characteristics in defining the treatment program. Three-dimensional self-correction is determined not only by the scoliotic pattern deviation but also by the patient’s performance capacity. Initially, the patient performs simple exercises while keeping a simplified self correction. Progressively, with the refinement of performance capacity and control, self-correction will become increasingly more complex until the optimal execution is achieved.

The choice of exercises follows the same pattern. Exercises become more and more difficult. The level of difficulty must always be adapted to the patient’s ability, and it must increase proportionally with the improvement of his ability.

Another characteristic of the SEAS protocol is that exercises change according to the different treatment phases. For example, if the patient is wearing a brace, the exercises are aimed to increase the mobility and plasticity of the torso and the spine, while allowing the brace to achieve the best possible corrective result.

According to SOSORT Guidelines treatment lasts until the risk of progression is eliminated. Typically in medium degree curves (below 20-25°), that are the scoliosis treated with SEAS exercises alone, treatment stops at European Risser 3. In patients wearing a brace, usually treatment lasts longer: in this case SEAS exercises are stopped 3 months after weaning definitively the brace, to help the final spinal stabilization in the first months without the brace.

### Patient evaluation to choose exercises

To prepare a physioterapic exercises plan for the rehabilitation of a patient who has suffered a trauma or surgery intervention is definitely important to evaluate muscle strength and joint flexibility because the patient has probably made a period of immobility and the re-education program will aim to improve these basic functions. In case of conservative treatment for scoliosis the situation is not the same.

The function we want improve with SEAS is the ability to self-correct the spine: this function simply does not exist without scoliosis, and it is also completely the opposite of what the pathology is creating in the patient. Consequently, this function does not need to be tested at the beginning, while during treatment it is tested simply checking how the patient is able to perform it.

Similar is the situation with the training of the ability to preserve self-correction in time. This too is a totally new function, and we need to train the neuromotorial control and the coordination of the spine, together with the stability function in general.

Nevertheless, to apply the SEAS protocol, an accurate evaluation of the patient is needed and this will be the basis for the choice of the exercises. Scheduled tests aim:

- to collect useful information that helps to choose the treatment plan in order to improve the subject’s morphology;
- to identify those functions that might eventually be deficient (i.e. muscular elasticity, neuromotor control, muscle resistance, etc.) and to integrate the program with specific exercises;
- to be able to re-evaluate every function, to verify whether the deficit is improving and to adapt specific exercises;
- to highlight deficient functions, to be able to choose accordingly the best stability training exercises for that specific patient. The complete evaluation is repeated each year. If the first evaluation emphasizes specific impairments, tests to evaluate these abilities should be performed more often, at least once every three months.

In any case, these evaluation test of the strength and mobility are not the most important assessment for the SEAS approach and they don’t particularly affect the treatment. These tests are simple means to discover any deficit that will try to recover while aiming to improve and build up a good self-correction ability. They represent only a complement of the treatment and not the real goal. An example: there are treatment methods that assume that the muscle retraction of some muscles are the causes of spinal misalignment. For this reason, the specific aim of this treatment is the resolution of the shortening of these muscles. Also in our assessment we evaluate the muscle stiffness and if patients show this, we use some exercises to improve it. In this case, the aim is simple: the improvement of the general condition of the patient.

During treatment we need difficulties to challenge the maintainance of self-correction, to improve its performance, and to build new neuromotorial strategies in the patient. The evaluation allow to focus these training difficulties to be used for self-correction; in this way, in the meantime the exercises improve the slight functional problems of strength, or balance, or shortening of muscles of the patient. But this is not the ultimate goal.

## ScoliosisManager

A very useful tool in developing an adequate exercise program is a free software program, available online, at www.scoliosismanager.it. This software has been developed under the electronic platform used by ISICO for the management of its patients. The name of the software, however, could be deceptive as to its potential use. The program was initially created to offer to physiotherapists, particularly those less experienced in the management of patients with spinal deviations, an active aid in composing an effective program of exercises. This is possible thanks to the accessible user interface that allows preparation of an exercise program under the guidance of the tool. Indeed, ScoliosisManager boasts a very large exercise database (more than thousand exercises) appropriate for patients affected by any type of musculoskeletal problems.

## Learning the SEAS approach

SEAS has no copyright and teachers are being trained around the world to make it known to physiotherapists as much as possible. Nevertheless, good training is mandatory; in fact, SEAS superficially appears easy, but real expertise on scoliosis, exercises, and patient and family management is required. Only adequate training and supervised practice will allow these skills to be achieved.

The regular SEAS course consists of two theoretical and practical sessions. In the first session, lasting three days, the principles of the technique are taught and the participants are provided with the tools to start the use of the technique with patients. In the second session, to be carried out not less than six months later, the SEAS concepts are deepened and mainly it consists in the supervision of the work that course participants have performed, discussing a series of clinical cases, chosen from their daily clinical practise.

## Results in the scientific literature

Different studies published or presented in occasion of international meetings over the past several years have provided a series of essential results detailing the SEAS approach as applied in different phases of treatment: hey include prospective [43-51] (Table 1) and retrospective studies [52-55] (Table 2), and case reports [56] (Table 3). Recently a randomised controlled trial has been published by an Italian group independent from the developers of SEAS [42]: the authors did not declare to have used the SEAS approach, presumably because it is the most diffused in Italy and considered as a standard [57]. Nevertheless, according to the description proposed in the text, they clearly used the SEAS active self-correction, as well as stabilisation and functional exercises, and a cognitive-behavioural approach according to SEAS principles [42]. This RCT has been supported by SOSORT as methodologically and technically well done [57]; this paper has shown the efficacy of specific scoliosis exercises according to the SEAS school in curves below 25° in avoiding progression and bracing. This paper added to the previous evidence reported in Table 1 shows the characteristics and results of SEAS exercises.

**Table 1:** **Research performed on SEAS approach: prospective studies**

**Paper**	**Topic**	**Design**	**Population**			**Results**
Negrini S et al. [43]	Efficacy of PSSE in AIS short-term	Prospective Controlled Cohort	Treatments	SEAS	UP	1 year Braced: SEAS 1 = UP 5 (P = 0.07) °C: ↑ SEAS (P < 0.05) Clinical results: SEAS > UP (P < 0.05)
			Patients	23	28	
			Females	18	19	
			Pathology	AIS		
			Age	12.7 ± 2.2	12.1 ± 1.1	
			°C	15.5 ± 5.4°	14.9 ± 6.0°	
			Other	No differences at baseline		
Negrini S et al. [44]	Efficacy of PSSE in AIS short-term	Prospective Controlled Cohort	Treatments	SEAS	UP	1 year Braced: SEAS 6.1% > UP 25.0% (P < 0.05) °C: SEAS ↑ UP SEAS: 23.5% ↑, 11.8% ↓ (excluded braced) UP: 11.1% ↑, 13.9% ↓ (excluded braced)
			Patients	35	39	
			Females			
			Pathology	AIS		
			Age	12.4 ± 2.2		
			°C	15.0 ± 6.0°		
			Others	No differences at baseline		
Romano M et al. [45]	Efficacy of PSSE in AIS medium term	Prospective Controlled Cohort	Treatments	SEAS	UP	2 years Braced: SEAS 10% > UP 27.6% (P < 0.05)
			Patients	20	37	
			Females			
			Pathology	AIS		
			Age	12.7 ± 2.2	12.19 ± 3	
			°C	15.3 ± 5.4		
			Others	ATR: 8.9 ± 2.8°		
Romano M et al. [46]	Efficacy of PSSE in AIS 10-20° short-term	Prospective Controlled Cohort	Treatments	SEAS	UP	End of growth (Risser 3) RR UP vs SEAS 1.74 (IC95 1.22-2.26)
			Patients	101	187	
			Females	190		
			Pathology	AIS		
			Age	0ver 10 years		
			°C	curves range 10-20°		
			Others	Risser 0–3 ; No differences at baseline		
Negrini S et al. [47]	Efficacy of PSSE in AIS	Prospective Controlled Cohort	Treatments	SEAS	UP NOE	End of Growth (Risser 3) °C - Intent-to-Treat Analysis RR NOE < SEAS 1.51 (IC95 1.21-1.80) (P < 0.05) RR NOE < UP 1.40 (IC95 1.08-1.72) (P < 0.05) RR UP = SEAS (P = NS) TRACE: ↑ SEAS (1.8) (P < 0.05); ↑ UP (1.5) (P < 0.05); SEAS > NOE (P < 0.05)
			Patients	145	148	
			Females			
			Pathology	AIS		
			Age			
			°C			
			Others	No differences at baseline		
Negrini S et al. [48]	Efficacy of PSSE in AIS in preparation to bracing	Prospective Controlled Cohort	Treatments	SEAS	UP	First x-ray without brace at 5 months °C: SEAS > UP Clinical results (improvement >5°C, >2° ATR): ↑ SEAS (P < 0.05); ↑ UP (P < 0.05); SEAS > UP (P < 0.05)
			Patients	110		
			Females	34		
			Pathology	AIS		
			Age	13.5 ± 2.4		
			°C	31.1 ± 11.1		
			Other	No differences at baseline		
Zaina F et al. [49]	Efficacy of PSSE in AIS in brace weaning	Prospective Controlled Cohort	Treatments	SEAS UP	DIS NOE	End of treatment °C: ↓ DIS 3.9° (P < 0.05); ↓ NOE 3.1° (P < 0.05). UP > DIS (P < 0.05)
			Patients	39	DIS 19 NOE 10	
			Females			
			Pathology	AIS		
			Age	15.1 ± 1.0		
			°C	22.0 ± 8.0°		
			Others	No differences at baseline		
Romano M et al. [50]	Efficacy of PSSE in AIS for balance function short-term	Prospective Controlled Cohort	Treatments	SEAS	NOE NOR	1 year Balance function: SEAS > NOR > NOE (P < 0.05)
			Patients	20	NOE 20 NOR 150	
			Females			
			Pathology	AIS		
			Age			
			°C			
			Others	No differences at baseline		
Romano M et al. [51]	Efficacy of PSSE in hyperkyphosis	Prospective Controlled Cohort	Treatments	SEAS	UP	End of growth Outcome: Plumbline distances at C7 and L3 C7: SEAS ↑ 61 ± 12 to 39 ± 11 (P < 0.05); UP ↑ 54 ± 12 to 41 ± 11 (P < 0.05) L3: SEAS ↑ 47 ± 11 to 41 ± 13 (P < 0.05)
			Patients	18	22	
			Females	21		
			Pathology	Hyperkyphosis		
			Age			
			°C			
			Others	No differences at baseline		

PSSE: Physiotherapeutic Scoliosis-Specific Exercises; SEAS: SEAS (Scientific Exercises Approach to Scoliosis) exercises therapy; UP: Usual Physiotherapy; DIS: Discontinuous Exercises; NOE: No Exercises; NOR: normal subjects; AIS: Idiopathic Scoliosis in Adolescents; °C: Cobb degrees; ATR: Angle of Trunk Rotation measured through Bunnell Scoliometer in degrees; TRACE: TRACE (Trunk Aesthetic Clinical Evaluation) from 1 (best) to 12 (worst).

Statistics: RR: Relative Risk of failure; IC95: 95% Confidence Interval; ITT: Intent-to-Treat Analysis.

Results: ↑: improved; ↓: worsened (progressed); >: better than; =: no differences; braced: number of braced patients; Clinical results: improvement of at least 5° Cobb, 2° ATR.

**Table 2:** **Researches performed on SEAS approach: retrospective studies**

**Paper**	**Topic**	**Design**	**Population**			**Results**
Negrini S et al. [52]	Efficacy of PSSE in AIS	Retrospective Controlled Cohort	Treatment	SEAS	UP	End of growth (Risser 3) °C: SEAS ↑ > UP ↓ (P < 0.05) ATR: SEAS ↑ (P < 0.05) Hump: SEAS ↑ (P < 0.05)
			Patients	33	89	
			Females			
			Pathology	AIS		
			Age	13.8 ± 3.1		
			°C	15.8 ± 11.9°		
			Others	ATR 5.6 ± 3.1°		
Romano M et al. [53]	Efficacy of PSSE in AIS	Retrospective Controlled Cohort	Treatment	SEAS	UP	End of growth (Risser 3) Braced: SEAS 28% > UP 43% (P < 0.05) Brace hours: SEAS > UP (P < 0.05) ATR: SEAS > UP (P < 0.05) TRACE: SEAS > UP (P < 0.05)
			Patients	78	98	
			Females			
			Pathology	AIS		
			Age			
			°C	14.7°		
			Others	ATR 6.3° - No differences at baseline		
Romano M et al. [55]	Efficacy of sport associated with PSSE in AIS	Retrospective Controlled Cohort	Treatment	SEAS Sport	SEAS No Sport	End of growth (Risser 3) °C =
			Patients	88	56	
			Females	497		
			Pathology	AIS		
			Age			
			°C	14.8 ± 5.7	16.6 ± 13.1	
			Others	No differences at baseline		
			Treatment	SEAS + Brace Sport	SEAS + Brace No Sport	End of growth (Risser 3) °C: No Sport ↑ 3.87° > Sport ↑ 3.01° (P < 0,05).
			Patients	182	217	
			Females			
			Pathology	AIS		
			Age			
			°C	32.2 ± 10.7	34.2 ± 13.2	
			Others	No differences at baseline		
Negrini A et al. [54]	Efficacy of PSSE in adults with progressive scoliosis	Retrospective Uncontrolled Cohort	Treatment	SEAS		3.5 years of treatment (range 1–24) °C: ↑ from 51° to 47° (P < 0.05).
			Patients	31		
			Females	28		
			Pathology			
			Age	38.0 ± 11.0 years		
			°C	51 ± 12°		
			Others			

PSSE: Physiotherapeutic Scoliosis-Specific Exercises; SEAS: SEAS (Scientific Exercises Approach to Scoliosis) exercises therapy; UP: Usual Physiotherapy; DIS: Discontinuous Exercises; NOE: No Exercises; NOR: normal subjects; AIS: Idiopathic Scoliosis in Adolescents; °C: Cobb degrees; ATR: Angle of Trunk Rotation measured through Bunnell Scoliometer in degrees; TRACE: TRACE (Trunk Aesthetic Clinical Evaluation) from 1 (best) to 12 (worst).

Statistics: RR: Relative Risk of failure; IC95: 95% Confidence Interval; ITT: Intent-to-Treat Analysis.

Results: ↑: improved; ↓: worsened (progressed); >: better than; =: no differences; braced: number of braced patients; brace hours: number of hours per day of bracing prescribed.

**Table 3:** **Researches performed on SEAS approach: case reports**

Negrini A et al. [55]	Efficacy of PSSE in adult scoliosis short-term	Case report	Treatment	SEAS	1 year ↑ from 47° to 28.5°
			Patient	1	
			Female	1	
			Pathology	AIS	
			Age	25 years	
			°C	47°	
			Others	Progressed 10° in 6 years	

PSSE: Physiotherapeutic Scoliosis-Specific Exercises; SEAS: SEAS (Scientific Exercises Approach to Scoliosis) exercises therapy; °C: Cobb degrees.

Results: ↑: improved.

Other key papers include the demonstration of the radiographic effectiveness of SEAS active self-correction [4], the importance of the Team Approach [58], the effectiveness of the cognitive-behavioural approach integrated with SEAS exercises [59], the importance of SEAS exercises associated with bracing [60].

## Conclusion

Beyond all the technical aspects presented in this paper, SEAS is an approach to scoliosis exercise treatment that differentiates from many others because of two main specificities: continuous improvement and development, to maintain its evidence base through the research results of the authors and the other conservative experts working on relevant topics; modern neurophysiological basis to treatment, so as to reduce requirements for patients (from a minimum of 90 to a maximum of 135 minutes of treatment per week) and costs for the family (one expert physiotherapist session every three months). This also makes it possible to treat a large number of patients by few expert physiotherapists, increasing their expertise to the maximum. Treatment planning has been optimized over the years so as to allow patients coming from far away to be treated: in fact in Italy while we regularly treat patients coming for years every six months from all over Europe, but also from other continents (including Australia).

Even if SEAS appears simple by requiring less physiotherapist supervision and by using fewer home exercises prescribed at a lower dose than some of the other scoliosis-specific exercise approaches, real expertise in scoliosis, exercises, and patient and family management is required.

### Consent

Written informed consent was obtained from the patients for the publication of this report and for any accompanying images.

This paper does not describe an experimental research. The approval of an ethics committee is not necessary. All the papers collected for the references have followed the original necessary approval.

## References

[1] Mollon G, Rodot JC (1986). Scolioses structurales mineures et kinèsitherapie. Etude statistique compareative des rèsultas. Kinesithérapie Scientifique.

[2] Stagnara P, Mollon G, de Mauroy JC (1978). Rééducation des scolioses. Expansion scientifique.

[3] Stagnara P (1985). Les déformations du rachis.

[4] Negrini A, Negrini S, Romano M, Verzini N, Parzini S, Monticone M (2006). A Blind Radiographic Controlled Study on the Efficacy of Active Self-Correction According to SEAS. 02. 3rd International Conference on Conservative Management of Spinal Deformities: 7–8 April 2006 2006.

[5] Negrini S, Atanasio S, Negrini A, Negrini A, Negrini A (2007). The Evidence-Based ISICO Approach to Spinal Deformities.

[6] Hodges PW (2003). Core stability exercise in chronic low back pain. Orthop Clin North Am.

[7] MacDonald DA, Moseley GL, Hodges PW (2006). The lumbar multifidus: does the evidence support clinical beliefs?. Man Ther.

[8] Smania N, Picelli A, Romano M, Negrini S (2008). Neurophysiological basis of rehabilitation of adolescent idiopathic scoliosis. Disabil Rehabil.

[9] Henschke N, Ostelo RW, van Tulder MW, Vlaeyen JW, Morley S, Assendelft WJ (2010). Behavioural treatment for chronic low-back pain. Cochrane Database Syst Rev.

[10] Ranganathan R, Newell KM (2013). Changing up the routine: intervention-induced variability in motor learning. Exerc Sport Sci Rev.

[11] Krakauer JW, Mazzoni P (2011). Human sensorimotor learning: adaptation, skill, and beyond. Curr Opin Neurobiol.

[12] Stokes IA, Geoffrey Burwell R, Dangerfield PH (2006). Biomechanical spinal growth modulation and progressive adolescent scoliosis-a test of the’vicious cycle’pathogenetic hypothesis: Summary of an electronic focus group debate of the IBSE. Scoliosis.

[13] Stokes IA, Spence H, Aronsson DD, Kilmer N (1996). Mechanical modulation of vertebral body growth: implications for scoliosis progression. Spine.

[14] Negrini S, Minozzi S, Bettany-Saltikov J, Zaina F, Chockalingam N, Grivas TB (2010). Braces for idiopathic scoliosis in adolescents. Spine (Phila Pa 1976).

[15] Negrini S, Minozzi S, Bettany-Saltikov J, Zaina F, Chockalingam N, Grivas TB (2010). Braces for idiopathic scoliosis in adolescents. Cochrane Database Syst Rev.

[16] Zaina F, De Mauroy JC, Grivas T, Hresko MT, Kotwizki T, Maruyama T (2014). Bracing for scoliosis in 2014: state of the art. Eur J Phys Rehabil Med.

[17] Romano M, Minozzi S, Bettany-Saltikov J, Zaina F, Chockalingam N, Kotwicki T (2012). Exercises for adolescent idiopathic scoliosis. Cochrane Database Syst Rev.

[18] Romano M, Minozzi S, Zaina F, Saltikov JB, Chockalingam N, Kotwicki T (2013). Exercises for adolescent idiopathic scoliosis: a Cochrane systematic review. Spine (Phila Pa 1976).

[19] Bettany-Saltikov J, Parent E, Romano M, Villagrasa M, Negrini S (2014). Physiotherapeutic scoliosis-specific exercises for adolescents with idiopathic scoliosis. Eur J Phys Rehabil Med.

[20] Stokes IA, Gardner-Morse M (2004). Muscle activation strategies and symmetry of spinal loading in the lumbar spine with scoliosis. Spine.

[21] Negrini S (2008). Bracing adolescent idiopathic scoliosis today. Disabil Rehabil Assist Technol.

[22] Negrini S, Marchini G, Tessadri F (2011). Brace technology thematic series – The Sforzesco and Sibilla braces, and the SPoRT (Symmetric, Patient oriented, Rigid, Three-dimensional, active) concept. Scoliosis.

[23] Weiss HR, Negrini S, Hawes MC, Rigo M, Kotwicki T, Grivas TB (2006). Physical exercises in the treatment of idiopathic scoliosis at risk of brace treatment – SOSORT consensus paper 2005. Scoliosis.

[24] Weiss HR (2011). The method of Katharina Schroth - history, principles and current development. Scoliosis.

[25] Rigo M, Quera-Salvá G, Villagrasa M, Ferrer M, Casas A, Corbella C (2008). Scoliosis intensive out-patient rehabilitation based on Schroth method. Stud Health Technol Inform.

[26] Dobosiewicz K, Durmala J, Kotwicki T (2008). Dobosiewicz method physiotherapy for idiopathic scoliosis. Stud Health Technol Inform.

[27] Maruyama T, Takeshita K, Kitagawa T (2008). Side-shift exercise and hitch exercise. Stud Health Technol Inform.

[28] Białek M (2011). Conservative treatment of idiopathic scoliosis according to FITS concept: presentation of the method and preliminary, short term radiological and clinical results based on SOSORT and SRS criteria. Scoliosis.

[29] Sanchez DJ, Reber PJ (2013). Explicit pre-training instruction does not improve implicit perceptual-motor sequence learning. Cognition.

[30] Sanli EA, Patterson JT, Bray SR, Lee TD (2012). Understanding self-controlled motor learning protocols through the self-determination theory. Front Psychol.

[31] Stokes IA, McBride C, Aronsson DD, Roughley PJ (2011). Intervertebral disc changes with angulation, compression and reduced mobility simulating altered mechanical environment in scoliosis. Eur Spine J.

[32] Aronsson DD, Stokes IA (2011). Nonfusion treatment of adolescent idiopathic scoliosis by growth modulation and remodeling. J Pediatr Orthop.

[33] de Sèze M (2012). Cugy Pathogenesis of idiopathic scoliosis: a review. Ann Phys Rehabil Med.

[34] Wang WJ, Yeung HY, Chu WC, Tang NL, Lee KM, Qiu Y, Burwell RG, Cheng JC.Top theories for the etiopathogenesis of adolescent idiopathic scoliosis. J Pediatr Orthop. 2011 Jan-Feb;31(1 Suppl):S14-27 10.1097/BPO.0b013e3181f73c12 21173615

[35] Haumont T, Gauchard GC, Lascombes P, Perrin PP.Postural instability in early-stage idiopathic scoliosis in adolescent girls. Spine (Phila Pa 1976). 2011 Jun;36(13):E847-54 10.1097/BRS.0b013e3181ff5837 21304436

[36] Junghanns H, Schmorl G (1971). The human spine in health and disease.

[37] Pope MH, Panjabi M (1985). Biomechanical definitions of spinal instability. Spine (Phila Pa 1976).

[38] Beales DJ, O’Sullivan PB, Briffa NK (2009). Motor control patterns during an active straight leg raise in pain-free subjects. Spine (Phila Pa 1976).

[39] Panjabi MM (1992). The stabilizing system of the spine. Part II. Neutral zone and instability hypothesis. J Spinal Disord.

[40] Rizzolatti G, Craighero L (2004). The mirror-neuron system. Annu Rev Neurosci.

[41] Rizzolatti G, Fabbri-Destro M, Cattaneo L (2009). Mirror neurons and their clinical relevance. Nat Clin Pract Neurol.

[42] Monticone M, Ambrosini E, Cazzaniga D, Rocca B, Ferrante S (2014). Active self-correction and task-oriented exercises reduce spinal deformity and improve quality of life in subjects with mild adolescent idiopathic scoliosis. Results of a randomised controlled trial. Eur Spine J.

[43] Negrini S, Negrini A, Romano M, Verzini N, Negrini A, Parzini S (2006). A controlled prospective study on the efficacy of SEAS.02 exercises in preventing progression and bracing in mild idiopathic scoliosis. Stud Health Technol Inform.

[44] Negrini S, Zaina F, Romano M, Negrini A, Parzini S (2008). Specific exercises reduce brace prescription in adolescent idiopathic scoliosis: a prospective controlled cohort study with worst-case analysis. J Rehabil Med.

[45] Romano M, Negrini S, Zaina F, Negrini A, Parzini S (2007). Does quality of exercises affect results in adolescent idiopathic scoliosis treatment to avoid braces? SEAS results at two years. 4th SOSORT International Conference – Boston (USA) 2007. Scoliosis.

[46] Romano M, Negrini A, Parzini S, Donzelli S, Zaina F, Negrini S (2012). Adolescent with 10° to 20° Cobb scoliosis during growth: efficacy of conservative treatments. A prospective controlled cohort observational study. 8th SOSORT Meeting, Barcelona, 2011. Scoliosis.

[47] Negrini S, Donzelli S, Negrini A, Parzini S, Romano M, Zaina F (2014). End Growth Results of Exercise Treatment to Avoid Bracing in Adolescents With Idiopathic Scoliosis: A Prospective Cohort Controlled Study.

[48] Negrini S, Negrini A, Romano M, Verzini N, Negrini A, Parzini S (2006). A controlled prospective study on the efficacy of SEAS.02 exercises in preparation to bracing for idiopathic scoliosis. Stud Health Technol Inform.

[49] Zaina F, Negrini S, Atanasio S, Fusco C, Romano M, Negrini A (2009). Specific exercises performed in the period of brace weaning can avoid loss of correction in Adolescent Idiopathic Scoliosis (AIS) patients: Winner of SOSORT’s 2008 Award for Best Clinical Paper. Scoliosis.

[50] Romano M, Tavernaro M, Negrini S (2006). Adolescent Idiopathic Scoliosis and his correlation with balance function. Can we improve them with physical exercises?.

[51] Romano M, Negrini S, Atanasio S, Fusco C, Zaina F, Negrini S (2009). Efficacy of specific SEAS exercises for hyperkyphosis: end-growth results of a controlled prospective study. 5th SOSORT International Conference - Lyon (F) 2009. Scoliosis.

[52] Negrini S, Romano M, Negrini A, Parzini S, Zaina F, Atanasio S (2009). 5th SOSORT End of treatment results for SEAS exercises: a controlled retrospective study. International Conference - Athens (GR) 2008. Scoliosis.

[53] Romano M, Negrini A, Pizzetti P, Negrini S (2010). Efficacy of SEAS exercises in AIS treatment at the end of growth: a retrospective controlled study in 176 patients 7th SOSORT International Conference - Montreal (CAN) 2010. Scoliosis.

[54] Negrini A, Negrini S, Parzini S, Romano M, Zaina F, Atanasio S (2009). SEAS exercises revert progression of adult scoliosis; a retrospective long- term study - 5th SOSORT International Conference - Athens (GR) 2008. Scoliosis.

[55] Romano M, Negrini S (2013). Sports in association with specific exercises can achieve better results in controlling the evolution of scoliosis? 10th SOSORT International Conference - Milan (IT) 2012. Scoliosis.

[56] Negrini A, Parzini S, Negrini MG, Romano M, Atanasio S, Zaina F (2008). Adult scoliosis can be reduced through specific SEAS exercises: a case report. Scoliosis.

[57] Negrini S, Bettany-Saltikov J, De Mauroy JC, Durmala J, Grivas TB, Knott P (2014). Letter to the editor concerning: “active self-correction and task-oriented exercises reduce spinal deformity and improve quality of life in subjects with mild adolescent idiopathic scoliosis. Results of a randomised controlled trial” by Monticone M, Ambrosini E, Cazzaniga D, Rocca B, Ferrante S (2014). Eur Spine J; DOI:10.1007/s00586-014-3241-y. Eur Spine J.

[58] Tavernaro M, Pellegrini A, Tessadri F, Zaina F, Zonta A, Negrini S (2012). Team care to cure adolescents with braces (avoiding low quality of life, pain and bad compliance): a case–control retrospective study. 2011 SOSORT Award winner. Scoliosis.

[59] Negrini A, Donzelli S, Lusini M, Minnella S, Zaina F, Negrini S (2014). A Cognitive Behavioural Approach Allows Improving Brace Wearing Compliance: An Observational Controlled Retrospective Study With Thermobrace.

[60] Negrini S, Donzelli S, Lusini M, Zaina F (2012). Bracing can reduce high degree curves and improve aesthetics immediately after the end of growth. Final results of a retrospective case series. Stud Health Technol Inform.

